# Cucurbit[7]uril-mediated aqueous self-assembly and supramolecular functionalisation of selenium nanoparticles

**DOI:** 10.1039/d6sc05797g

**Published:** 2026-10-15

**Authors:** Daniel Ponsford, Si Yin Tee, Choon Peng Teng, Siew Lang Teo, Andrew Thomson, Yidan Yin, Ceri A. Foster, Gregory Q. Wallace, William J. Peveler, Ming-Yong Han, Tung-Chun Lee

**Affiliations:** a Department of Chemistry, University College London London WC1H 0AJ UK; b Institute for Materials Discovery, University College London London WC1E 7JE UK tungchun.lee@ucl.ac.uk; c Institute of Materials Research and Engineering (IMRE), Agency for Science, Technology and Research (A*STAR) Singapore 138634 Singapore; d School of Chemistry, University of Glasgow Joseph Black Building Glasgow G12 8QQ UK william.peveler@glasgow.ac.uk; e Institute of Molecular Plus, Tianjin University Tianjin 300072 China han_mingyong@tju.edu.cn

## Abstract

We report the first example of noncovalent binding interactions between cucurbit[7]uril (CB7, a synthetic molecular receptor) and a surface comprising a non-metallic elemental solid (selenium, Se). The interaction enabled the preparation of CB7-selenium nanocomposites with controllable morphologies *via* aqueous self-assembly. Colloidal Se nanomaterials have broad applications in human and animal health due to their excellent anti-oxidant, antimicrobial and chemotherapeutic properties. We demonstrate that chemical functionality can be readily introduced to the surface of selenium colloids functionalised with CB7, *via* host–guest complexation with guests of interest. In this instance, drug molecules were encapsulated within CB7 molecules at the selenium nanoparticle surface to produce functionalised CB7-Se NP nanoaggregates with enhanced antimicrobial and cytotoxic activity. Tuneable aggregation of non-metallic nanoparticles opens up new routes to functional hybrid nano-assemblies for safe and precise integration with biology. Meanwhile, our supramolecular approach is expected to facilitate rapid development of selenium-based nanomaterials and drug formulations *via* mix-and-match of guest molecules within the in-built molecular receptors.

## Abstract 2

Barrel-shaped cucurbituril non-covalently crosslinks nonmetallic selenium nanoparticles, creating functional nanoaggregates. The degree of aggregation is controllable, supramolecular activity is maintained and biological activity is demonstrated.

## Graphical abstract

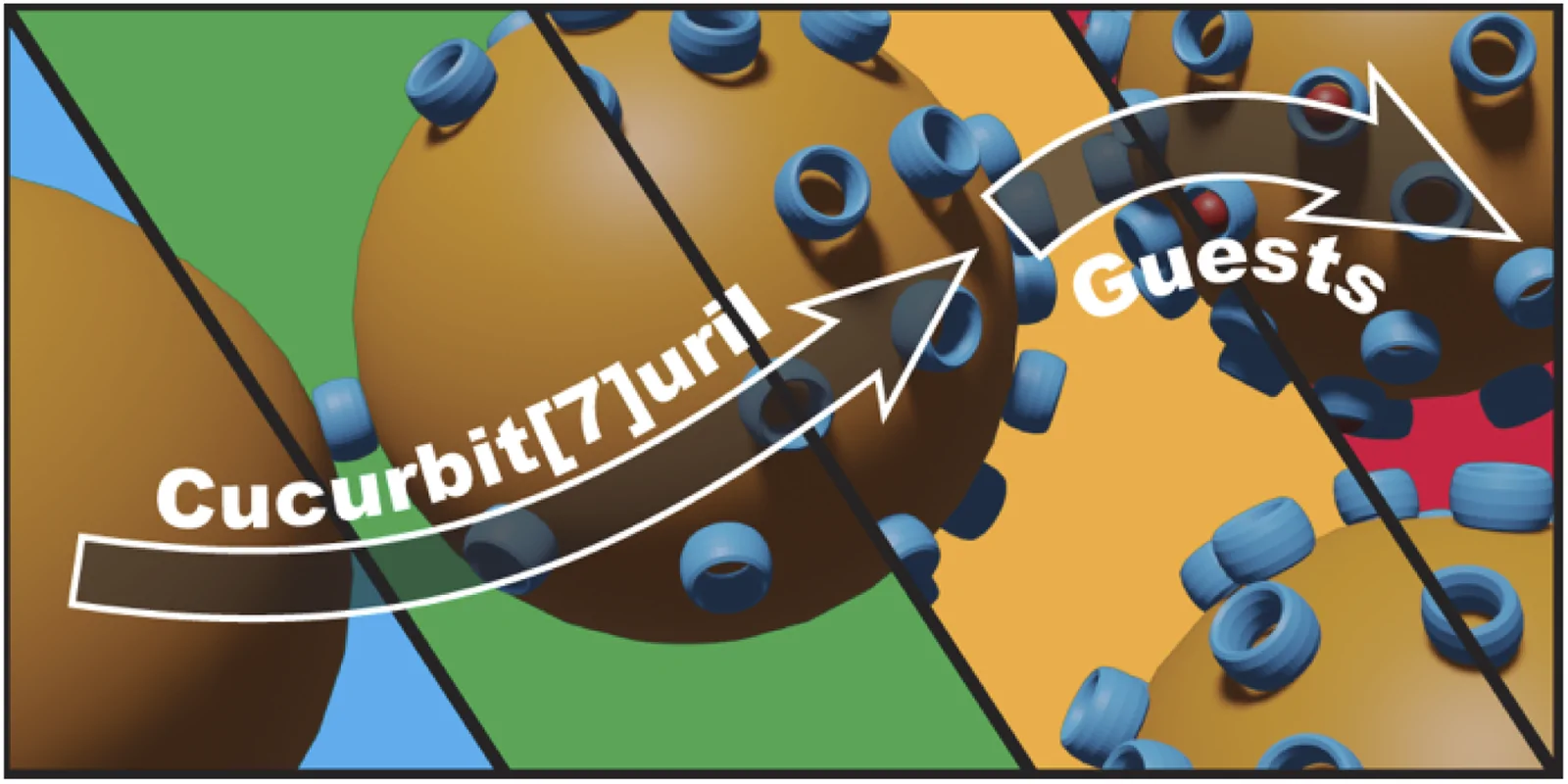

## Introduction

Selenium (Se) is an essential trace element in the human body, playing a crucial role in various physiological functions, including antioxidant defence and DNA synthesis.^1,2^ Although Se is crucial for maintaining healthy bodily function, it has a narrow window of positive effect (typically below 400 µg or 5 µmol a day), and small concentration increases can cause toxicity. Elemental selenium nanoparticles (Se NPs) are markedly less toxic than organic and inorganic selenium compounds whilst retaining antioxidant efficacy,^3^ and due to their form as a colloidal suspension and low concentration, safe dosage can be much more precisely controlled than that of bulk Se.^4^ Most studies of Se NPs concern their biomedical and therapeutic applications, such as in the treatment of oxidative stress and inflammatory disesases.^5–8^ In particular, Se NPs are notable for their ‘anticancer’ activity.^9,10^

Due to their native properties, Se NPs are attractive candidates for co-therapy strategies, acting as an innate chemotherapeutic whilst also enabling the delivery of drug molecules to target sites, as they are readily surface-loaded with drugs,^9^ and are known to hyper-accumulate in cancerous cells.^11^ However, Se NPs with their native coating from synthesis often exhibit poor colloidal stability, which leads to compromised efficacy and physicochemical characteristics.^4^ Se NPs are also of growing interest in biosensing thanks to their chemoresponsive and optoelectronic properties, but to achieve function, the surface must also be modified.^12,13^ As a consequence, Se NPs are typically functionalised in order to introduce finely tuneable properties for the specific application. Stabilisers previously explored include chitosan and other polysaccharides (*e.g.* hyaluronic acid),^14^ peptides and proteins including bovine serum albumin,^15^ and other polymers such as polyvinyl alcohol (PVA).^16^ The covalent modification of such polymeric ligands to add functionality is not always straightforward, and may reduce colloidal stability. Alternatively, the supramolecular functionalisation of nanoparticles, with host–guest systems in place on the surface represents a facile strategy for the conjugation of a variety of useful moieties to nanoparticles.^17^ Such surface bound macrocycles can act as carriers of cargo for drug delivery or used to attach secondary ligand shells *via* host–guest ‘supramolecular click chemistry’.^18–20^

Cucurbit[*n*]urils (CB*n*) are a family of macrocycles known for their rich supramolecular chemistry. The hydrophobic internal cavities of CB*n* are able to accommodate a vast range of guest molecules with high binding affinities (including some of the strongest known host–guest interactions),^21,22^ whilst the hydrophilic carbonyl-fringed portals are excellent receptors for ion binding,^23^ surface attachment,^24^ and nanoparticle capping.^25,26^ Importantly, the cavity remains available for the encapsulation of suitably sized guest molecules even as CB*n* are bound to surfaces or ions *via* their portals.^27,28^ In addition, the encapsulation of drug molecules by related macrocycles has been shown to confer protection to the drug from chemical, physical and enzymatic degradation, and to enhance the solubility of hydrophobic drugs.^29^

While CB*n* are known to bind to nanoparticle surfaces of metals (*e.g.* Au, Ag, Pd),^30,31^ oxides (*e.g.* Fe_2_O_3_),^32^ silver bromide,^33^ and II–VI semiconductors (*e.g.* CdTe, ZnS)^18,34^*via* their electron-rich portals, their direct interactions with non-metallic elemental materials remain far more poorly studied. Herein, we explored the interactions between cucurbit[7]uril (CB7) and Se NPs (a non-metallic element). We exploited such noncovalent binding interactions to generate a series of self-assembled structures in aqueous media, ranging from quasi-fractal NP networks to colloidally stable nanoaggregates, through controlling the colloidal aggregation kinetics. The CB7-Se NPs can be further functionalised by utilising the host–guest complexation of CB7 (Fig. 1). We exemplify this novel approach for stabilising and functionalising Se NPs in a rapid, facile and effective manner, and including functional guests on the Se NP surface. In particular, 5-fluorouracil (FU) is incorporated to synergistically combine the therapeutic and bioaccumulation properties of Se NPs with the cytotoxic and chemotherapeutic effects of FU in cell-death assays.^9^

**Fig. 1:**
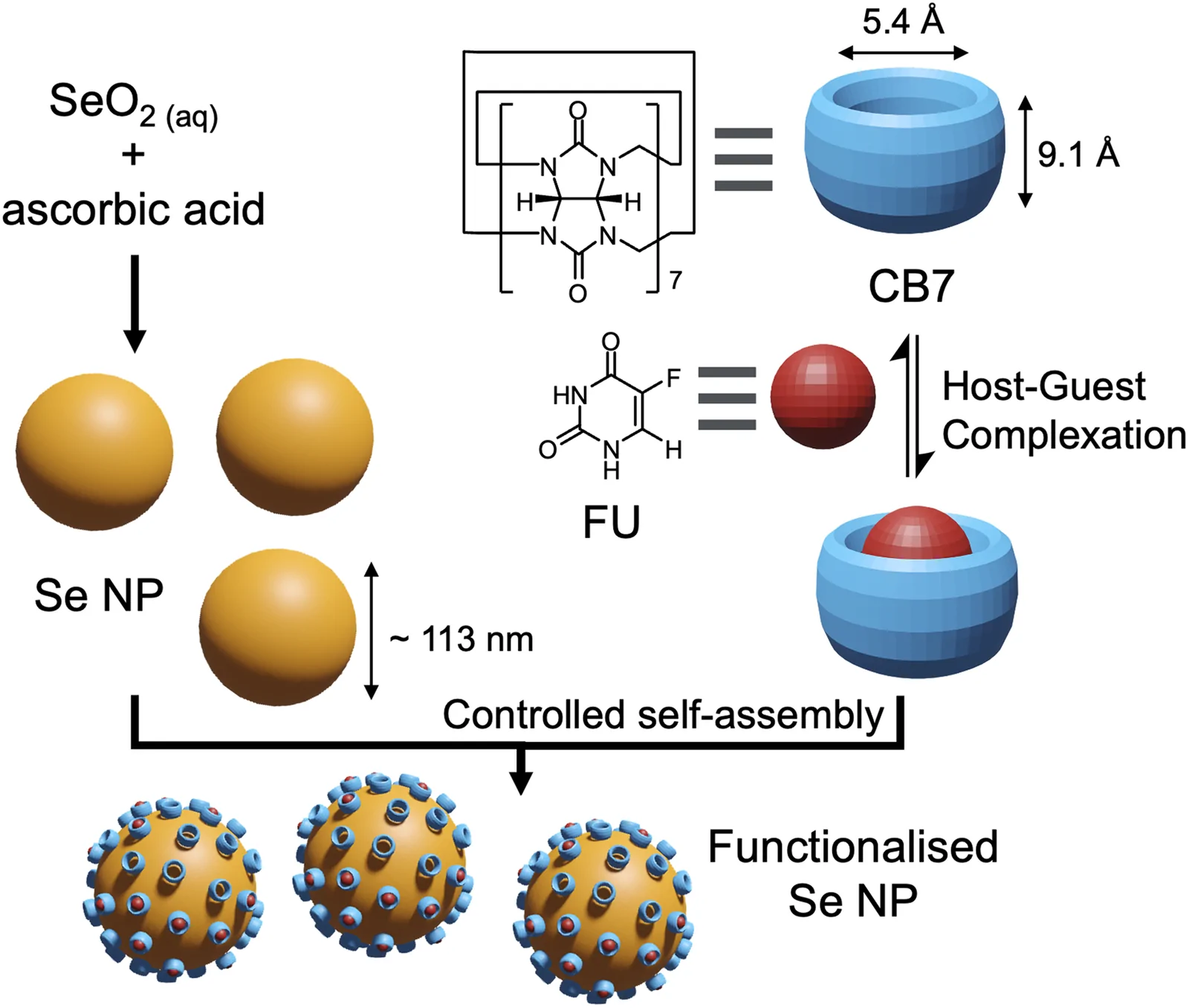
Supramolecular functionalisation of Se NPs (orange spheres) *via* hierarchical aqueous self-assembly mediated by CB7 (blue toroids). Encapsulated moieties (red spheres, for example fluorouracil or FU here), which can provide additional functionality and/or stabilisation to the nanoparticles, are first encapsulated into the cavity of CB7 *via* host–guest complexation and subsequently loaded onto the surface of Se NPs through novel noncovalent interactions between the CB7's portal and the Se NPs. CB7 has a height of 9 Å, with an inner rim diameter of 5.4 Å and outer diameter of 16 Å.

## Results and discussion

### Se NPs can be surface-modified with CB7

Ascorbate-stabilised, amorphous Se NPs were synthesised *via* the reduction of aqueous selenous acid (0.5 mM) by aqueous l-ascorbic acid (7 mM) as per modified literature procedures,^35^ to form an orange colloidal solution (Fig. 2a) in deionised (DI) water. The orange colouration is consistent with amorphous Se, as confirmed by X-ray diffraction (Fig. S1). Moreover, we performed transmission electron microscopy (TEM) to characterise the nanoscale morphology of the Se NPs (Fig. 2b). The Se NPs were predominantly spherical, with an average diameter of 113 ± 6 nm. Dynamic light scattering (DLS) measurements of the Se NPs in DI water gave a hydrodynamic diameter of 118 nm, in excellent agreement with the TEM data. By assuming all Se in the precursor was converted into Se NPs, the concentration of Se NPs is estimated to be *ca.* 20 pM, *i.e.* 1.2 × 10^10^ NPs per mL.

**Fig. 2:**
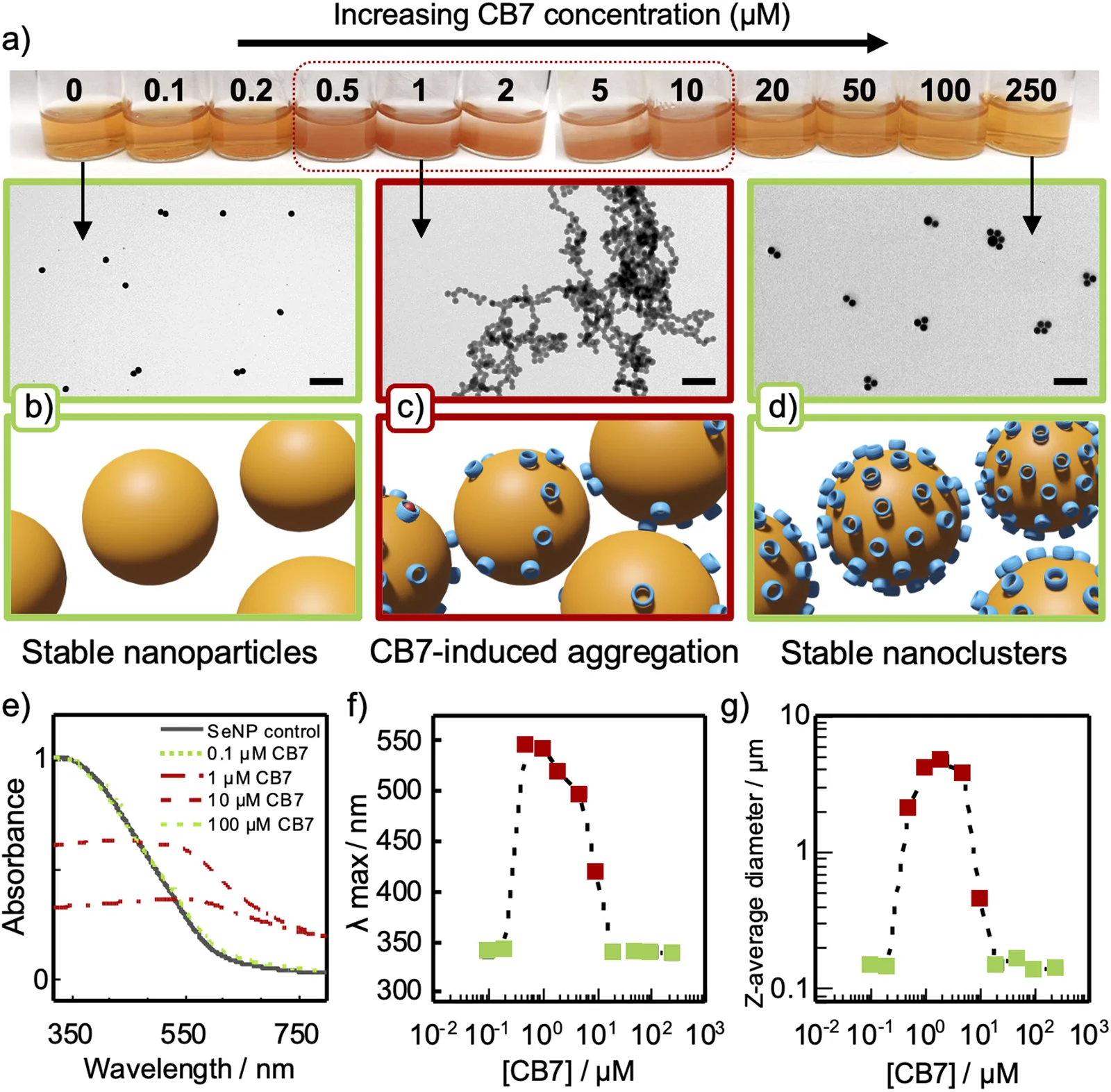
Characterisation of CB7-Se NPs. (a) Photos of colloidal Se NP solutions with increasing concentration of CB7. (b)–(d) representative TEM images corresponding to selected samples (scale bars = 0.5 µm) and corresponding schematics illustrating the morphology of the nanoaggregates of CB7-Se NPs. Not drawn to scale. (e) UV-vis absorption spectra of selected CB7-Se NP samples. (Additional UV-vis in Fig. S7) effects of CB7 concentration on (f) the wavelength at maximum absorption in UV-vis spectra (*λ*_max_), and (g) intensity-weighted hydrodynamic diameters (Z-average) of CB7-Se NP samples measured by DLS (both recorded 3 hours after CB7 addition).

The orange colloidal solution of Se NPs displayed a strong, broad absorption peak at *ca.* 350 nm (Fig. 2a–e) as a shoulder on the expected ∼250 nm absorbance, which is in good agreement with calculated values for Se NPs with a diameter of approximately 110 nm.^36,37^ A Tauc plot (Fig. S2) extracted from this UV-vis data indicated a band gap of 1.97 eV, also in good agreement with literature values for amorphous selenium (a direct bandgap semiconductor).^38^ The zeta potential of the Se NPs was measured as −22 mV. This negative zeta potential results from ascorbate anions from the synthesis weakly adsorbed to the Se NP surface.

To investigate the interactions between CB7 and Se NPs, various amounts of CB7 (final concentrations between 0.1–250 µM) were added to the as-synthesised Se NPs in colloidal form (∼40 µg mL^−1^ Se), to generate CB7-Se NPs. CB7 was chosen because of its excellent water solubility and larger cavity volume. Interestingly, we observed non-monotonic dependence of aggregation behaviours on increasing concentration of CB7. In particular, CB7-Se NPs with a low concentration of CB7 (<0.5 µM) were colloidally stable for more than 72 hours and did not exhibit any observable difference to the control sample of Se NPs (Fig. 2a). However, as CB7 concentration was increased to between 0.5–10 µM, aggregation and sedimentation became noticeable to the naked eye after *ca*. 3 hours, as well as a slight darkening of colour, suggesting Se NPs being “glued” together *via* two-portal binding of CB7 to form large aggregates. Crucially, as CB7 concentrations were further increased (20–250 µM), sedimentation was no longer observed, suggesting that the colloid returned to a metastable state of individual nanoparticles or small nanoaggregates, where the surface becomes saturated with CB7 that hinders two-portal binding, as illustrated by the schematics in Fig. 2b–d. Similar concentration dependence of aggregation behaviours can be observed in the well-established case of CB7-Au NPs (Fig. S3).^30^

TEM was performed to characterise the nanoscale morphology of the CB7-Se NPs at different CB7 content, and to provide further evidence for the above hypothesis (Fig. 2b–d). At very low concentrations of the macrocycle, the majority of CB7 are doubly capped by Se NPs, and trapped in the form of NP-dimers or trimers. As the CB7 concentration increases, additional CB7 can form supramolecular cross-links between dimers and other oligomers of NPs, resulting in the rapid formation of extended quasi-fractal networks at a critical CB7 concentration of *ca.* 1 µM (Fig. 2c), resembling diffusion-limited colloidal aggregation (DLCA) kinetics (Fig. S4). Upon a further increase in CB7 concentration, these aggregates begin to break up, as excess CB7 molecules cap the surface of Se NPs rather than bridging them (Fig. 2d), resulting in the formation of clusters of Se NPs *via* a slower reaction-limited colloidal aggregation (RLCA) route.^26,39^ The disaggregation of CB7 capped Se NPs, upon reaching a critical ratio of CB7 per NP, opens up the possibility of performing host–guest chemistry on the surface of these nanoparticles and is explored further below.

In order to quantify the effects of CB7 concentration on aggregation behaviours, we performed ensemble measurements in solution using UV-vis spectroscopy (Fig. 2e and f) and dynamic light scattering (DLS) measurements (Fig. 2g) on the CB7-Se NPs. A plot of the wavelength at maximum absorption (*λ*_max_) *versus* CB7 concentration (Fig. 2f) highlights the drastic increase in nanoparticle aggregation between 0.2 and 0.5 µM CB7, and a more gradual change with increasing CB7 concentration thereafter. At macrocycle concentrations of 20 µM and above, the UV-vis absorption spectra are not markedly different to that of the control (0 µM), and the CB7-Se NPs remain stable in solution. The observed aggregation-induced red shift of *λ*_max_ can be attributed to changes in scattering by larger particles (Mie theory) and interparticle energy transfer within the nanoaggregates, which indicates the narrow spacing between the Se NPs, consistent with our previous observations of CB7-CdTe quantum dots systems.^37^ The critical CB7 concentration at which extended quasi-fractal networks form can also be illustrated by DLS data shown in Fig. 2g (size distribution data by intensity and kinetics data are given in Fig. S5 and S6). The CB7-Se NP aggregates with the largest hydrodynamic diameter were recorded at a CB7 concentration of 2 µM. In excellent agreement with the UV-vis data, individual Se NPs were stable in solution at CB7 concentrations of 0.2 µM and below, and small CB7-Se NPs nanoaggregates at concentrations of 20 µM and above. Interestingly, the threshold of stable nanoaggregates (20 µM CB7) corresponds to a 50–60 fold excess of CB7 with respect to the total available surface binding sites on Se NPs, consistent with our expectation that such noncovalent binding interaction is relatively weak, and therefore an excess of CB7 is needed to quantitatively displace the competitive ligands (*i.e.* ascorbate anions) while minimising the formation of cross links (see SI for detailed calculations).

Further characterisation was conducted to reveal the binding orientation of CB7 on the surface of Se NPs and the nature of the binding interactions. Interparticle spacing between Se NPs was measured and compared based on dried samples using the TEM. In the absence of CB7, the average distance between neighbouring Se NPs was measured as 3.1 ± 0.9 nm, according to line profiles (Fig. S8). In the presence of CB7, the average interparticle distances were found to be 1.8 ± 0.4 nm at 1 µM CB7, corresponding to a visibly aggregated state, and 3.5 ± 0.4 nm at 250 µM CB7, when the solution appears to be disaggregated. These averaged distances suggest that, at low CB7 concentration, a significant portion of the nanojunctions between Se NPs are bridged by a single CB7 molecule (portal-to-portal van der Waals distance = *ca.* 1 nm) with the portals facing the Se NP surface. Meanwhile, at high CB7 concentration, the Se NPs tend to be spaced by two or three CB7 molecules upon drying, as the Se NP surfaces are saturated with CB7. Moreover, a narrower range of interparticle spacings, as indicated by a smaller standard deviation, was observed in the presence of CB7 (Fig. S9), suggesting the presence of attractive interactions between CB7 (a rigid macrocyclic molecule) and Se NPs that precisely hold the Se NPs in close proximity. We do acknowledge that there might be systematic error in the spacing as measured by the TEM due to the thick Se NPs and beam damage at higher magnification. In particular, for spherical NPs of *ca.* 100 nm, the closest point of contact between the two NPs is *ca.* 50 nm above the carbon membrane of the TEM grid, making it challenging to obtain the accurate focus. In addition, there is an increased flux of scattered electron generated by the thick Se NPs which decreases the effective spatial resolution of the measurement. Nevertheless, the overall trend of interparticle spacings provides preliminary evidence of CB7 binding onto the surface of Se NPs *via* its electron-rich portals.

We utilised Fourier-transform infrared (FTIR) spectroscopy, in order to obtain specific insights into the chemical bonding and microenvironments around the carbonyl groups of the CB7 portals upon binding to Se NPs. The FTIR spectra confirmed the presence of CB7 in the 250 µM CB7-Se NP samples after centrifugal washing. In comparison with pure CB7, the absorption peak of the CB7 carbonyl group in CB7-Se NPs was blue-shifted by approximately 15 cm^−1^, indicating that the C=O bond becomes stiffer upon binding to the Se NPs (Fig. 3a and b). The observed bond stiffening can be attributed to the confined microenvironment on the NP surface and/or the strengthening of the C=O bond *via* induction effects of the diffused electron clouds on Se.

**Fig. 3:**
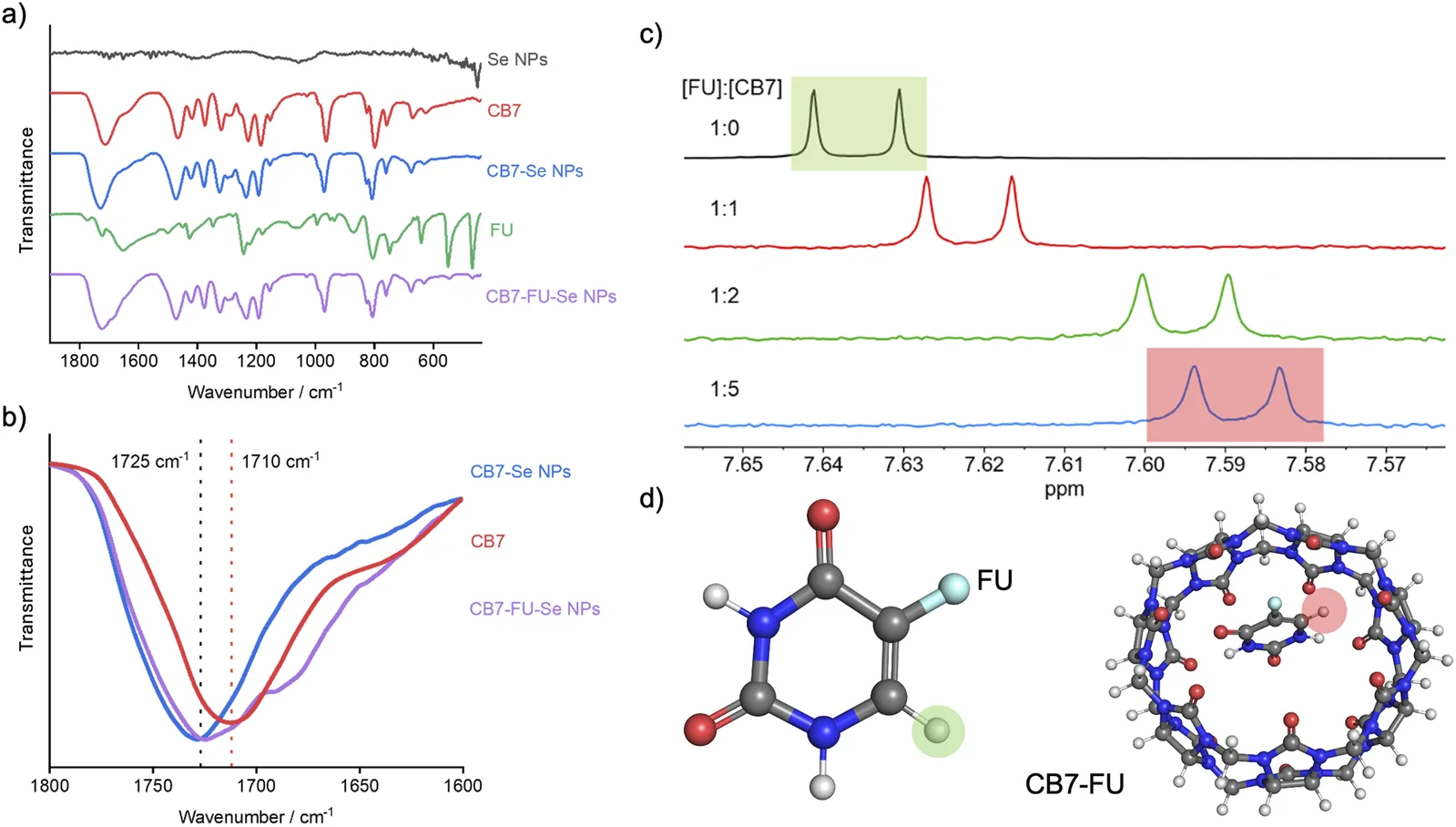
FTIR spectra. (a) Se NP, CB7 and FU (and composites thereof) samples. The composite samples were 250 µM CB7 and FU in each case. After centrifugation, the supernatant was removed and the samples redispersed in water (in order to remove excess ascorbic acid and CB7). (b) The blue-shift in the CB7 C=O peak of the composite samples, relative to pure CB7. (c) ^1^H NMR spectra of free and complexed FU (D_2_O, 500 MHz). An FU concentration of 0.3 mM was maintained in each case, and the [FU] : [CB7] ratios are given above the NMR spectra. Full NMRs given in Fig. S11. (d) DFT-optimised models of free FU and a CB7-FU complex. The proton of FU and the corresponding ^1^H NMR signals are marked by green and red highlights for free and CB7-complexed FU, respectively.

To elucidate the nature of the binding interactions, in particular, whether (1) CB7 directly binds to the Se NP surface *via* displacing the weakly adsorbed ascorbate anions or (2) CB7 (or CB7 with a bound proton in the portal) binds to the ascorbate layer of the Se NPs *via* electrostatic interactions, we have performed zeta potential measurements on as-synthesised Se NPs and CB7-Se NPs. Upon addition of 10 µM CB7, the zeta potential of the nanoparticles changed from −22 mV to +37 mV (Fig. S10) and the zeta potential remained above +30 mV with increasing CB7 concentration. The significant increase in the absolute value of zeta potential suggests that CB7-Se NP nanoclusters formed at high CB7 concentrations exhibit enhanced colloidal stability, consistent with the visual observations and the UV-vis and DLS results (Fig. 2). Moreover, the notable charge reversal on the surface of Se NPs, induced by CB7, supports the scenario where CB7 directly binds onto the NP surface and displaces previously adsorbed ascorbate anions. Meanwhile, the distal CB7 portals either bind positive ions (*e.g.* H^+^), or induce positive dipoles on Se^0^, in a noncovalent fashion, leading to a reversal in zeta potential, as also observed in CB*n*-Au NPs.^30^ Interestingly, despite the different chemistry, selenium and gold exhibit very similar electronegativity (2.55 and 2.54 on the Pauling scale, respectively) that could rationalise the noncovalent interactions between CB7 and Se. We also note that the aggregation of Se NPs is not due to depletion forces, which would be expected to monotonically increase in magnitude with increasing CB7 concentration.

### CB7 bound on the surface of Se NPs can encapsulate small molecules *via* host–guest binding

In order to assess the potential for functionalisation of CB7-capped Se NPs, the encapsulation of 5-fluorouracil (FU) by CB7 was investigated by ^1^H nuclear magnetic resonance (^1^H NMR) spectroscopy and NP-CB7-FU interactions were validated with IR spectroscopy (Fig. 3) as well as functional testing. FU was chosen for this initial investigation because it is a representative small drug (a pyrimidine analogue) with chemotherapeutic (cytotoxic) properties, where co-delivery with Se could act as a sensitiser to the chemotheraputic or a co-operative agent.^9^

In all NMR experiments an FU concentration of 0.3 mM was maintained whilst CB7 concentration was varied. A distinct upfield shift for the doublet corresponding to the CH proton of FU was observed upon the addition of increasing amounts of CB7 (Fig. 3c and S11). Such upfield shift can be attributed to the shielding effects of the CB7 molecular framework, and thus verifying that FU is indeed encapsulated deep inside the cavity of CB7. Moreover, upon increasing concentration of CB7, the FU peaks were observed to broaden relative to those of free FU, revealing that the host–guest binding kinetics is in the fast-exchange regime of the 500 MHz NMR timescale. The observed upfield peak shifts and peak broadening are consistent with the supramolecular encapsulation of guest molecules. The upfield shifted doublet represents the time-averaged signal of both complexed and free FU, indicating the reversible and dynamic nature of the encapsulation.

Molecular modelling was performed to gain further insights on the binding pose of FU inside CB7's cavity and to estimate the binding thermodynamics. FU was docked within CB7 by screening binding poses using AutoDock Vina's search algorithm and scoring function. Geometry optimisation and binding affinity calculations were carried out for each unique binding pose in Spartan 20 and cross validated with Gaussian 16 using density functional theory (DFT) at the wB97XD/6-31G* level of theory and a CPCM implicit solvent model. A dispersion-corrected hybrid functional wB97XD was employed to model the van der Waals interactions between the host and the guest.

The DFT-optimised binding pose confirmed that FU is fully encapsulated by CB7's cavity, consistent with the ^1^H NMR findings. The full encapsulation of FU will ensure the portals of CB7 remain unhindered and available for the binding to the Se NPs. The binding energy of the ground state CB7-FU complex was calculated to −23.09 kcal mol^−1^ (Fig. 3d and S12). Adding the contribution of thermochemical corrections to free energy yielded a binding constant log *K* of 6.30 (See SI for further technical details). This was larger than a value estimated from NMR titration of CB7-FU complexation (Fig. S13), but the equilibrium was found to be easily influenced by pH and other solvent effects.

Following these experiments, FU was pre-mixed with CB7 at a 1 : 1 ratio to form a host–guest complex, which was then added to Se NPs. We note that the aggregation kinetics did not exhibit any observable change, supporting the hypothesis that the FU is encapsulated deep inside the cavity, consistent with the NMR and molecular modelling findings. Furthermore, FTIR confirmed the presence of CB7-FU in CB7-FU-Se NP samples even after washing (Fig. 3a), with a similar blue-shift in the CB7 carbonyl peak of approximately 15 cm^−1^ (Fig. 3b). The zeta potential of the CB7-FU-Se NPs containing 50 µM CB7-FU was measured as +34.9 mV, also indicative of functionalised nanoparticles.

There is precedent for the formation of inclusion complexes and pseudorotaxanes at the surface of nanoparticles using host–guest chemistry of cyclodextrins (CD) and CB. Both βCD and MeβCD have been used to stabilise Se NPs previously with some evidence of inclusion complexes formed at the surface, using adamantyl-functionalised RNA,^40^ or the drug indomethacin.^41^ Cyclic peptides have previously been co-assembled on the surface with chemotherapeutic doxorubicin, although there is little evidence of host–guest interactions in this case,^42^ and a positively charged pillar[5]arene was used on mesoporous Se nanomaterials to putatively form an inclusion complex with methyl orange.^43^ Whilst CdTe and CdSe/ZnS nanoparticles have been previously modified with CB*n* and shown to form strong (two to three orders of magnitude stronger than CD-adamantyl) inclusion complexes,^18^ there are no reports for such phenomena on non-metallic elemental NPs, such as Se NPs.

To further explore the potential for inclusion complexes at the Se NP/CB7 surface, a study was undertaken with adamantyl triethylene glycol (A3OH), a molecule previously demonstrated to bind CB7 at the surface of CdTe NPs without displacing the macrocycle from the surface.^18^ Pre-aggregated Se NPs were more challenging to disaggregate with this good CB7 binder, likely due to the larger size and stronger van der Waals interactions between the NPs that were harder to offset with a short triethylene glycol spacer. However, at the right ratio aggregation could be slowed or prevented by pre-mixing the CB7 and A3OH (Fig. S14), and FTIR showed CB7 and A3OH was retained at the Se NP surface (Fig. S15), suggesting this could be a route to non-covalent surface functionalisation in future.

### CB7-FU-Se NPs display combined cytotoxic and antimicrobial activity

The potential for the noncovalently functionalised Se NPs in this study to impact microbe and cell viability was investigated, as a proof-of-concept application. An equimolar CB7-FU mixture was added to Se NPs synthesised as above, to generate CB7-FU-Se NPs with a final CB7 and FU concentration of 50 µM. A higher CB7-FU concentration was avoided to limit the amount of free CB7-FU (*i.e.* not attached to the surface of the Se NPs) in the system, whilst promoting the CB7 and FU to remain complexed. The CB7-FU-Se NPs mixture, and analogously prepared Se NPs, CB7-Se NPs and FU + Se NPs (no CB7), were then diluted to various selenium concentrations (between 0 and 40 µg mL^−1^ Se in the NPs).

The cytotoxic effects of these undecorated and decorated Se NPs against breast cancer-derived epithelial (MCF7) cells were assessed by a colorimetric cell counting kit-8 (CCK8) assay. Control experiments, without Se NPs but with equivalent CB7 and FU loadings were also performed to account for the effect of any free CB7-FU complex in solution (Fig. 4a and S16). Se NPs began to significantly reduce cell viability after 24 hours at Se concentrations above 5 µg mL^−1^ (above 60 µM Se and 6.25 µM CB7/FU), relative to the Se-free control (Fig. S16), highlighting the critical role of Se NPs in the cytotoxic activity. At these higher concentrations, Se NPs functionalised with CB7, and CB7-FU, were observed to drastically reduce cell viability compared to bare Se NPs or the mixture of Se NPs and FU. At concentrations of 10 and 20 µg mL^−1^, cell viability was most significantly reduced by Se NPs decorated with CB7-FU complexes. By comparison, gold nanoparticles functionalised with cyclodextrin-FU complexes required significantly higher concentrations or longer time periods to reduce cell viability to a similar extent.^44^ We speculate that the enhanced cytotoxicity of CB7-Se NPs and CB7-FU-Se NPs relative to Se NPs results from the change in zeta potential to positive, which facilitates increased cellular adhesion and uptake, and hence better Se and FU delivery.^45^

**Fig. 4:**
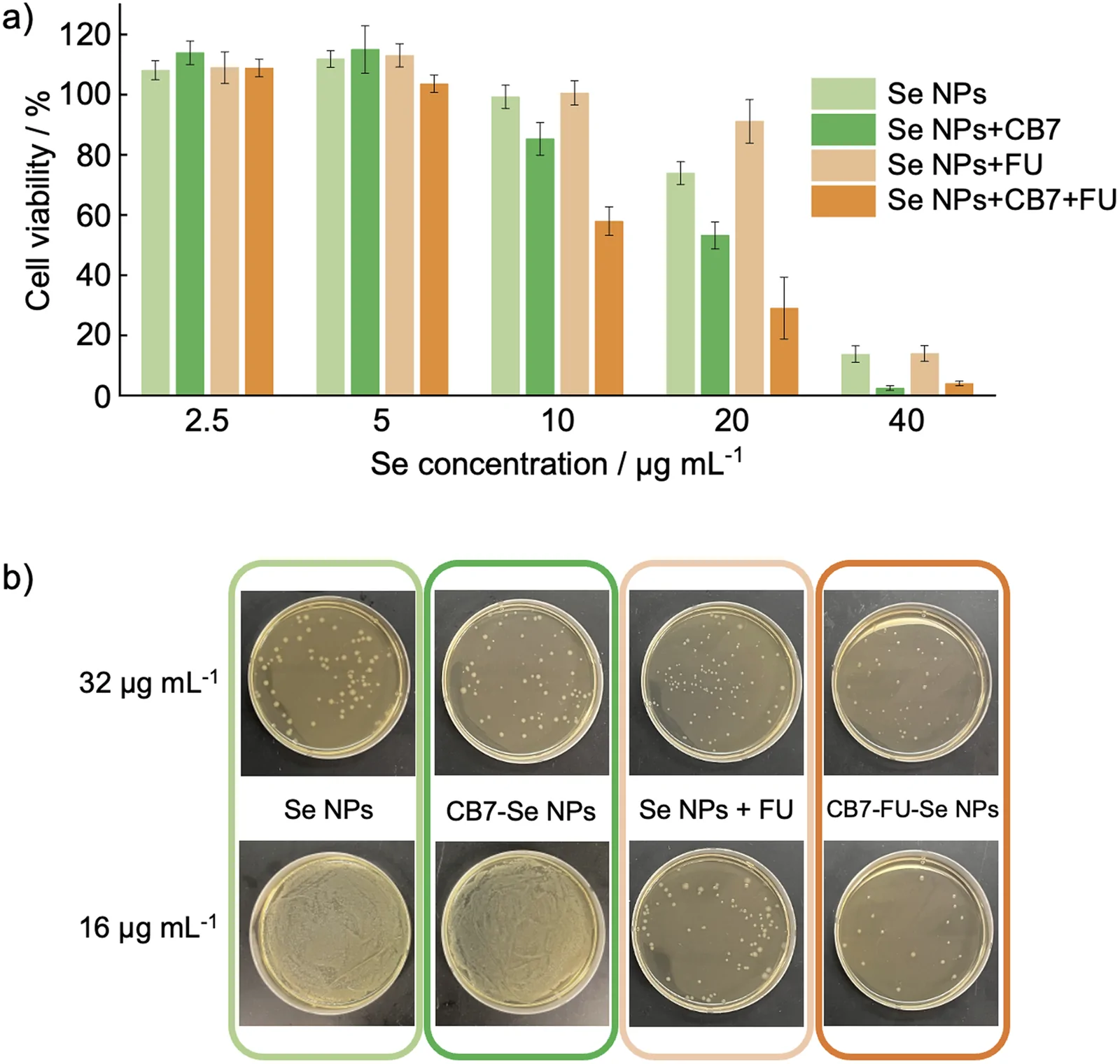
a) The cytotoxic impact of CB7-FU-Se NP composites on the cell viability of MCF7 cells after a 24 hour incubation period. Error bars are the standard deviation on 5 replicates. (b) The antimicrobial effects of the composites at higher (above) and lower (below) concentrations against *E. coli* on LB agar plates after 24 hours.

Se NPs have been previously conjugated directly with FU but the mechanism of physisorption was unclear.^9^ The supramolecular carrier approach here enables more diverse and varied applications, as well as a more stable conjugate. Previous work with βCD-Se NPs as carriers for RNA showed cell penetration was possible but the mechanism was not discussed.^40^ Direct decoration of Se NPs with a cell penetrating peptide and co-delivery with various cytotoxic compounds showed similar levels or less cell death as our work, but with much higher loading of Se NPs (50 µM).^42^

We also tested the system as an antimicrobial agent. The addition of CB7-FU also yielded nanoparticles with higher antimicrobial activity, as determined by a broth dilution method followed by plating the broths onto agar plates (Fig. 4b). The minimum inhibitory concentrations (MICs, Table S1) for both the FU-Se NPs and CB7-FU-Se NPs, with respect to *E.Coli*, were 16 µg mL^−1^, half that of the corresponding samples without FU. These MIC values are comparable to those of Se NPs with potent antimicrobial properties which have been previously reported.^46^ As colonies were observed on the agar plates, these suggests that the *E. Coli* were only inhibited (bacteriostatic). Overall, CB7-FU-Se NP composites exhibited the highest cytotoxicity and the strongest antimicrobial activity among the samples tested, likely due to the synergistic effects of FU delivery and the reversal of the Se NP surface charge to positive.

These findings act as proof-of-concept for the surface decoration and functionalisation of Se NPs *via* host–guest chemistry and demonstrate utility in improving the efficacy of chemotherapeutics for human and bacterial cells. The colorimetric assay used in this instance reveals global growth information, and so additional tests will be necessary to elucidate the specific mechanisms of cell delivery and death triggered by functionalised Se NPs, as well as the role of each component in the enhanced antimicrobial activity of Se NPs loaded with FU. Work continues to this end.

## Conclusion

We discovered noncovalent binding interactions between cucurbit[7]uril and selenium, which represent the first example of CB*n* directly binding to the surface of a non-metallic element. This binding interaction was subsequently harnessed to prepare a range of CB7-selenium nanocomposites with diverse morphology, from colloidally stable nanoparticle oligomers to extended quasi-fractal assemblies, through controlled aqueous self-assembly. The binding was revealed to be mediated *via* the electron-rich carbonyl portal of CB7, as evidenced by the shift of the C=O stretch signal in the FTIR spectrum and the charge reversal effects of the CB7-Se NPs upon increasing concentration of CB7. Furthermore, chemical functionality can be readily introduced to these colloids *via* host–guest complexation with guests of interest. In this instance, drug molecules (5-fluorouracil) were encapsulated within CB7 molecules to afford CB7-FU-Se NPs with enhanced antimicrobial and cytotoxic activity. Antimicrobial and cytotoxicity studies highlighted the significant differences between bare Se NPs and those functionalised by CB7, and the strong action of functionalised Se NPs against cancerous epithelial cells in preliminary experiments, as measured by cell growth inhibition.

It is notable that in addition to its biological properties, Se is a semiconductor that exhibits piezo- and thermoelectric properties and a nonlinear optical response, and is thus an important photoconductive material suited to a variety of applications including (photo)catalysis and xerography.^47–49^ Therefore, we expect that the highly tuneable and modular nature of this supramolecular functionalisation motif should enable the development of a vast array of functional selenium nanoparticles, for uses as therapeutic agents, precursors for structured selenium-based thermoelectric materials, and antimicrobial coatings (given the amorphous nature of these nanoparticles) – and the ability to further modify the Se NP surface *via* host–guest chemistry (the so-called ‘hook and eye’ or ‘supramolecular click’ approach) opens up exciting new directions in modular materials chemistry. We anticipate further studies of the system with ^77^Se NMR and saturation-transfer difference or other applicable NMR techniques may further enhance our understanding of the surface chemistry. Beyond selenium, our findings could also motivate future searches for noncovalent interactions between CB*n* and a range of allotropes of non-metallic elements, *e.g.* tellurium and sulphur, as well as inspire novel design of hybrid nanomaterials *via* CB*n*-mediated co-assembly of nanoparticles of different material compositions.

## Author contributions

Conceptualization: DP, MYH, TCL; data curation: DP, YY, CAF, GQW, WJP, TCL; formal analysis: DP, WJP, TCL; funding acquisition: WJP, MYH, TCL; investigation: DP, SYT, CPT, SLT, AT, YY, CAF, GQW; methodology: DP, YY, WJP, MYH, TCL; project administration: WJP, MYH, TCL; resources: DP, SYT, CPT, SLT, AT, YY, WJP, MYH, TCL; supervision: SYT, WJP, MYH, TCL; visualization: DP, CAF, GQW, WJP, TCL; writing – original draft: DP, WJP, TCL writing – review & editing: All authors.

## Conflicts of interest

There are no conflicts of interest to declare.

## Supplementary Material

SC-OLF-D6SC05797G-s001

## Data Availability

The data supporting this article have been included as part of the supplementary information (SI). Supplementary information: additional methodology, full characterisation data including XRD, electron microscopy, spectrocopy and light scattering measures, as well as additional calculations. See DOI: https://doi.org/10.1039/d6sc05797g.
