# Supplementary material for: Cucurbit[7]uril-mediated aqueous self-assembly and supramolecular functionalisation of selenium nanoparticles
Source: Chem Sci. 2026 Oct 15. Online ahead of print. doi: 10.1039/d6sc05797g (PMC13691645; doi:10.1039/d6sc05797g)
Supplement: Supplementary file 1 [file SC-OLF-D6SC05797G-s001.pdf]

Supplementary Information (SI) for Chemical Science.
This journal is © The Royal Society of Chemistry

Electronic Supplementary Information

Cucurbit[7]uril-mediated aqueous self-assembly and supramolecular functionalisation of selenium nanoparticles

Daniel Ponsford,[abc] Si Yin Tee,[c] Choon Peng Teng,[c] Siew Lang Teo,[c] Andrew Thomson,[ab] Yidan Yin,[ab] Ceri A. Foster,[d] Gregory Q. Wallace,[d] William J. Peveler,[d]* Ming-Yong Han,[e]* Tung-Chun Lee[ab]*

a. Department of Chemistry, University College London, London, WC1H 0AJ, UK

b. Institute for Materials Discovery, University College London, London, WC1E 7JE, UK
E-mail: tungchun.lee@ucl.ac.uk

c. Institute of Materials Research and Engineering (IMRE), Agency for Science, Technology and Research (A*STAR) Singapore, 138634, Singapore

d. School of Chemistry, Joseph Black Building, University of Glasgow, Glasgow, G12 8QQ, UK.
E-mail: william.peveler@glasgow.ac.uk

e. Institute of Molecular Plus, Tianjin University, Tianjin 300072, China
Email: han_mingyong@tju.edu.cn

Contents

Materials and Methods 2
- Chemicals 2
- Se NP synthesis and modification 2
- X-ray diffraction (XRD) 2
- Transmission electron microscopy 2
- UV-visible absorption spectroscopy 3
- FTIR 3
- Dynamic light scattering (DLS) and zeta potential measurements 3
- Nuclear magnetic resonance spectroscopy 3
- Estimation of nanoparticle concentration and surface area 3
- Computational modelling 3
- Minimum inhibitory concentration and antimicrobial activity evaluation 4
- Cytotoxicity evaluation 4

Additional data 4
- Fig S1: Se NP phase determination 4
- Fig. S2 Tauc plots for bandgap determination 5
- Fig. S3 Comparative kinetics data for CB7-mediated gold nanoparticle aggregation 5

Fig. S4 UV-visible kinetics 6
Fig. S5-S6 Dynamic light scattering..6
Fig S7 Additional UV-visible spectroscopy...7
Fig. S8-S9 Particle diameter/interparticle distance measurements 7
Fig. S10 Zeta potential measurements..9
Fig S11 Full CB7-FU NMR 9
Fig. S12 DFT modelling 10
Fig. S13 Additional NMR titrations 11
Fig. S14 Additional host-guest chemistry at Se NP surfaces..12
Fig. S15 Additional FTIR spectroscopy...14
Fig. S16 Additional cytotoxicity data 14
Table S1 Antimicrobial tests 15
References...15

Materials and Methods

Chemicals

Selenium dioxide and L-ascorbic acid were purchased from Sigma Aldrich and used as received. Cucurbit[7]uril was prepared according to the literature and multiple different batches were used.[1] Adamantyl triethylene glycol (A3OH) was synthesized according to previous reports.[2] Human breast adenocarcinoma cells (MCF7) and *Escherichia coli (E. coli*, MG1655) were obtained from ATCC. Penicillin-streptomycin and phosphate buffered saline (PBS) were purchased from Thermofisher Scientific while Luria-Bertani (LB) broth and agar were bought from Invitrogen. Cell counting kit-8 (CCK8) assay was purchased from Dojindo Laboratories. All the other agents were used as received.

Se NP synthesis and modification

Aqueous selenium dioxide (0.1 mL, 25 mM) was diluted in deionised water (3.9 mL), and 1 mL aqueous L-ascorbic acid (35 mM) was quickly added under stirring (~400 rpm), to give a final concentration of 0.5 mM Se and 7 mM L-ascorbic acid respectively. The solution was stirred at room temperature for 20 minutes.

Aqueous CB7 (2 mM stock solution), or a 1:1 aqueous CB7:5-fluorouracil (FU) solution (2 mM stock solution), were then added at various ratios to the selenium nanoparticles as formed above, as described in the main text.

X-ray diffraction (XRD)

XRD patterns were recorded using a Bruker D8 Advance X-ray diffractometer operating at 40 kV and 40 mA Cu Kα radiation (λ = 1.5418 Å). As-synthesised Se NP samples were drop cast onto glass slides and dried under ambient conditions before XRD patterns were collected.

Transmission electron microscopy

Transmission electron microscopy (TEM) images were recorded on a JEOL-2100 transmission electron microscope using an acceleration voltage of 200 kV. Samples were prepared by applying one drop of

the as-synthesised cucurbituril-selenium nanoparticle composites onto a lacey formvar/carbon 300-mesh Cu grid.

UV-visible absorption spectroscopy

Absorption spectra were recorded using a Shimadzu UV-1800 double-beam UV-Vis-NIR spectrophotometer at room temperature and quartz cuvettes.

FTIR

Fourier-transform infrared spectra were measured in the range 400 – 2000 cm^{-1}, for samples drop-cast onto aluminium foil and air-dried, on a PerkinElmer Spectrum 2000 FTIR spectrometer.

Dynamic light scattering (DLS) and zeta potential measurements

Particle size and zeta potential were recorded on a Malvern Zetasizer Nano ZS. A 633 nm He-Ne laser light source and an avalanche photodiode (APD) detector were used. Zeta potential measurements were recorded in a dip cell with parallel Pd electrodes.

Nuclear magnetic resonance spectroscopy

Nuclear magnetic resonance (NMR) spectra were recorded on a JEOL JNM-ECA500II spectrometer at 500 MHz, or Bruker AVIII HD 400 MHz, using D_2O as a solvent and internal reference at 4.79 ppm. Figures were processed using MestReNova software.

Estimation of nanoparticle concentration and surface area

Nanoparticle concentrations were estimated assuming complete consumption of the 0.5 mM Se precursor into spherical vitreous Se nanoparticles, of density 4.28 g cm^{-3} and homogenous size. TEM sizing gave an estimate of 113 nm ± 6 nm, so the range of values are calculated here. CB7 is modelled as having a 2 nm^2 footprint and a packing density on a sphere of between 0.84 and 0.9 (perfect close packing), however this does not necessarily account for surface plateaus, edge effect etc.

Diameter (nm)	Volume (nm^3)	NP mass ($x10^{-15}$ g)	NPs L^{-1}	NP molar conc. (pM)	NP Surface Area (nm^2)	CB7 per NP (packing density 0.84 - 0.9)	CB7 conc for complete coverage (µM)
113 (mean)	755499	3.23	$1.22 x10^{13}$	20	40115	16848 -18052	0.34 -0.37
119 (+1SD)	882347	3.78	$1.05 x10^{13}$	17	44488	18685 -20020	0.32 -0.35
107 (-1SD)	641431	2.75	$1.44 x10^{13}$	24	35968	15107 -16186	0.36 -0.39

Given the relative size of the CB7 and the Se NP, the estimate for coverage does not vary hugely, and so 20 µM CB7 to disaggregate the NPs corresponds to a 50-60 fold excess.

Computational modelling

FU was docked within CB7 by screening binding poses using AutoDock Vina's scoring function.[3,4] Optimum binding location within the cavity was confirmed by geometry optimisation applying GFN2-xTB semiempirical tight binding.[5] Further geometry optimisation and binding affinity calculations were carried out in Spartan 20 and cross validated by Gaussian 16 at the wB97XD/6-31G* level of theory.[6] Restricted (close-shell) models were used in all DFT calculations. Refer to the "DFT modelling" section below for full technical details, additional data and discussions.

Minimum inhibitory concentration and antimicrobial activity evaluation

Se NP samples were first diluted with PBS to the following concentrations: 1.25, 2.5, 5, 10, 20, 40 µg/mL. 1 mL of the samples at each concentration was then incubated with 200 µL of *E.coli* suspension (5×10^5 CFU/mL) at 37 °C for 24 h. This was performed in triplicates. *E.coli* incubated with 1 mL of PBS was used as the control. The turbidity of the suspension was evaluated the next day. Minimum inhibitory concentration (MIC) was determined by the lowest concentration at which the incubated suspension remains visibly clear. The antimicrobial activity was further evaluated by plating 100 µL of the suspensions on LB agar plates and incubated at 37 °C overnight. Colony counting was performed the next day. Mean colony counts and standard deviation (SD) were calculated for each sample type and concentration. All plates were prepared at a 100-fold dilution factor.

Cytotoxicity evaluation

MCF7 cells were cultured in DMEM medium containing 10% FBS and 1% penicillin-streptomycin. 1×10^4 MCF7 cells were added to each well of a 96 well plate and incubated at 37°C overnight. 100 µL of the Se NP samples in the following concentrations (1.25, 2.5, 5, 10, 20, 40 µg/mL) was added to the wells the next day in 5 replicates. For the control wells, 100 µL of DMEM medium was added. After 24 h incubation, the medium was removed from each well. 100 µL of DMEM medium containing 10% CCK8 assay was then added to each well and incubated for 3 hours. The cell viability is quantified by measuring the absorbance of orange formazan (at 450 nm) which is produced when tetrazolium salt in the CCK8 assay is reduced in the presence of viable cells.

Additional data

Fig S1: Se NP phase determination

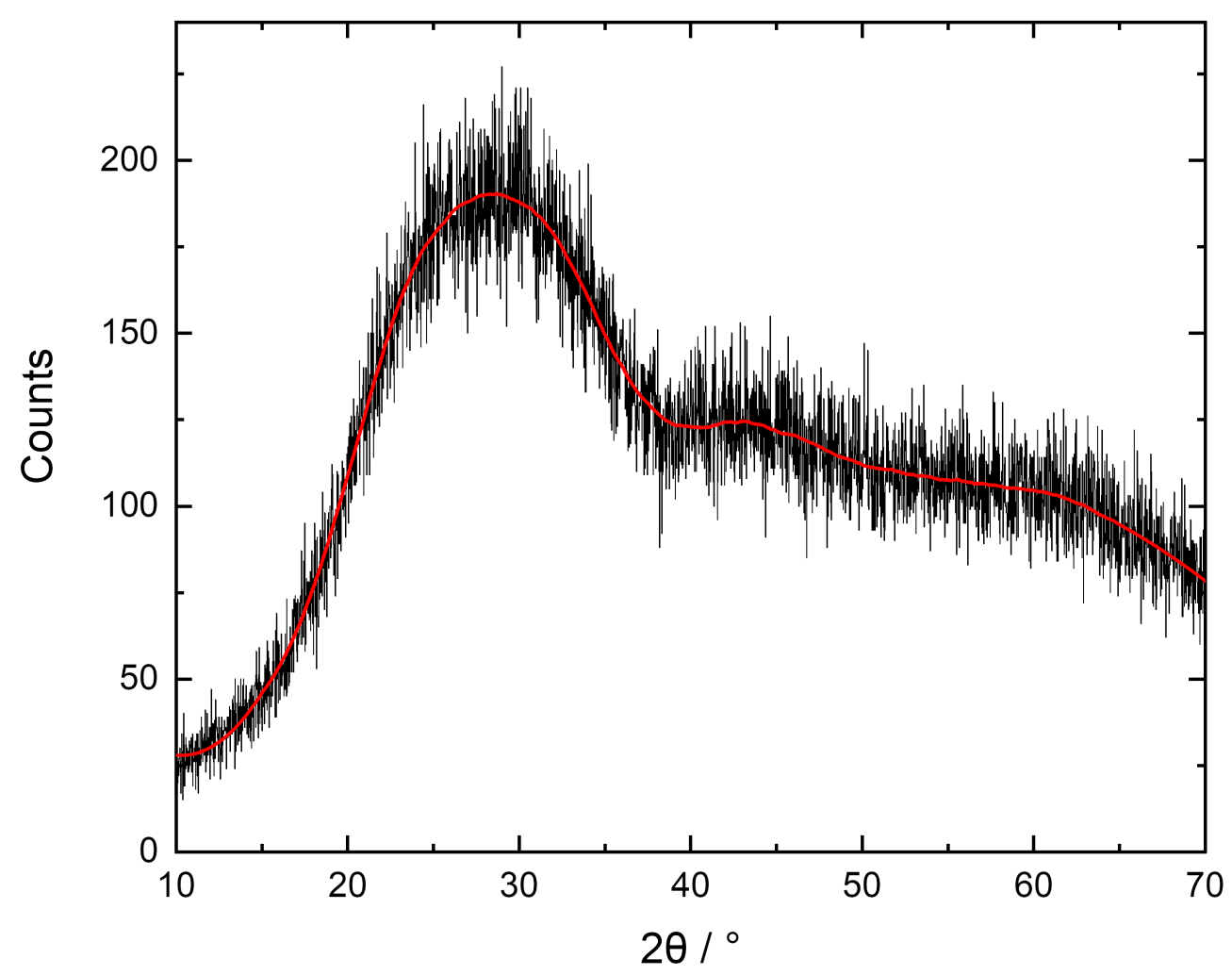

Fig. S1. XRD pattern of as-synthesised amorphous Se NPs.

Fig. S2 Tauc plots for bandgap determination

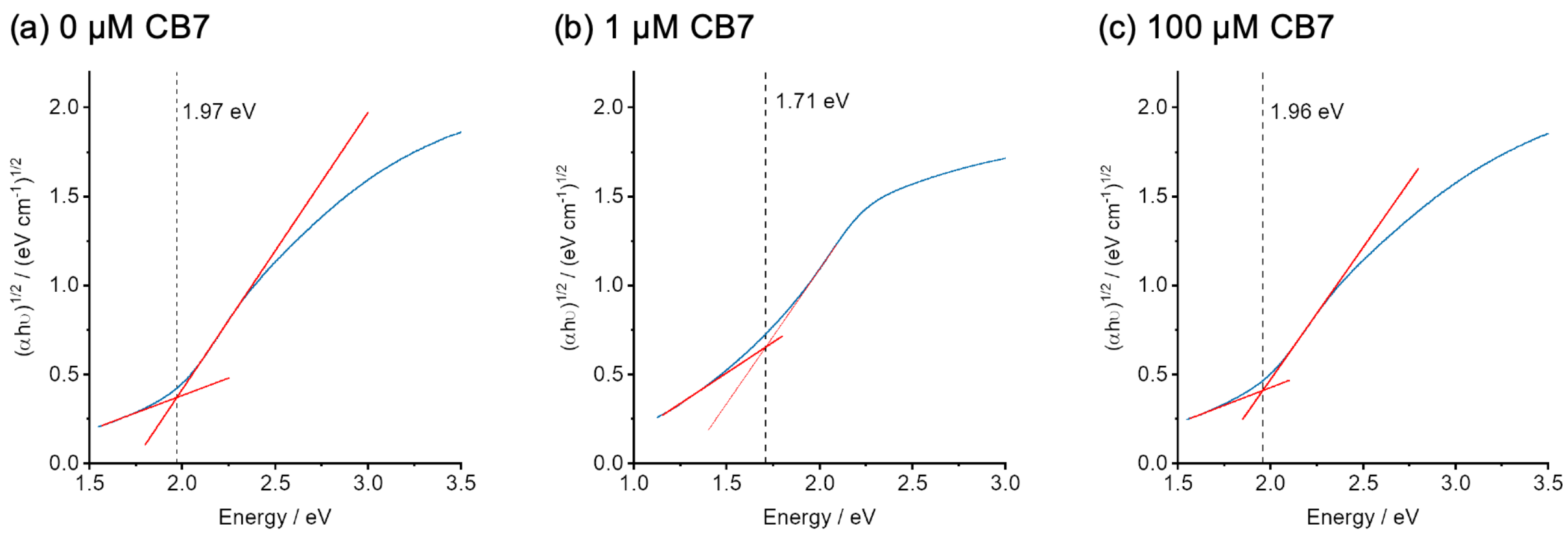

Fig. S2. Tauc plots derived from UV-vis spectra for the determination of the semiconductor bandgap for selenium. a) As-synthesised Se NPs, b) Se NPs + 1 µM CB7 and c) Se NPs + 100 µM CB7. Aggregation with CB7 (b) shows a distinct reduction of the observed band gap vs the pristine, or fully CB capped samples. However, we note accurate fitting of the band gap of aggregates is challenging due to the strong scattering of the sample.

Fig. S3 Comparative kinetics data for CB7-mediated gold nanoparticle aggregation

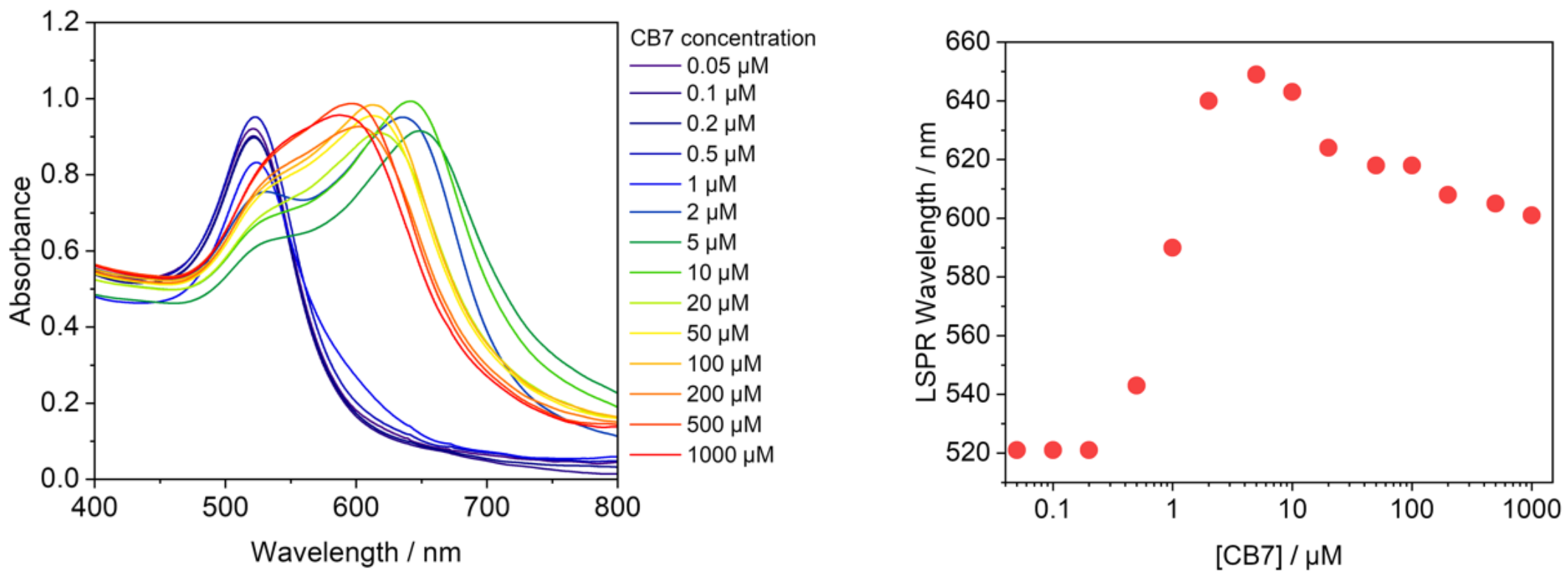

Fig. S3. a) UV-vis spectra of 20-nm gold nanoparticles (Au NPs) samples 30 minutes after the addition of CB7. b) LSPR wavelengths of Au NPs or CB7-Au NP nanoaggregates at CB7 concentrations from (a). At low CB7 concentrations, trace CB7 were trapped within oligomers of Au NPs while the majority of Au NPs exist as monomers. Beyond a threshold of ca. 0.5 µM CB7, aggregation becomes significant as evident by the red-shift of the LSPR wavelength due to plasmonic coupling between closely spaced Au NPs. The degree of aggregation reached a maximum at ca. 5 µM CB7 to form extended quasi-fractal assemblies. At high CB7 concentration, the CB7-Au NP formed nanoaggregates of smaller sizes, as indicated by the decreasing trend of the LSPR wavelength, due to the saturation of CB7 on the NP surface that subsequently hindered the same CB7 binding to and bridging two Au NPs.

Fig. S4 UV-visible kinetics

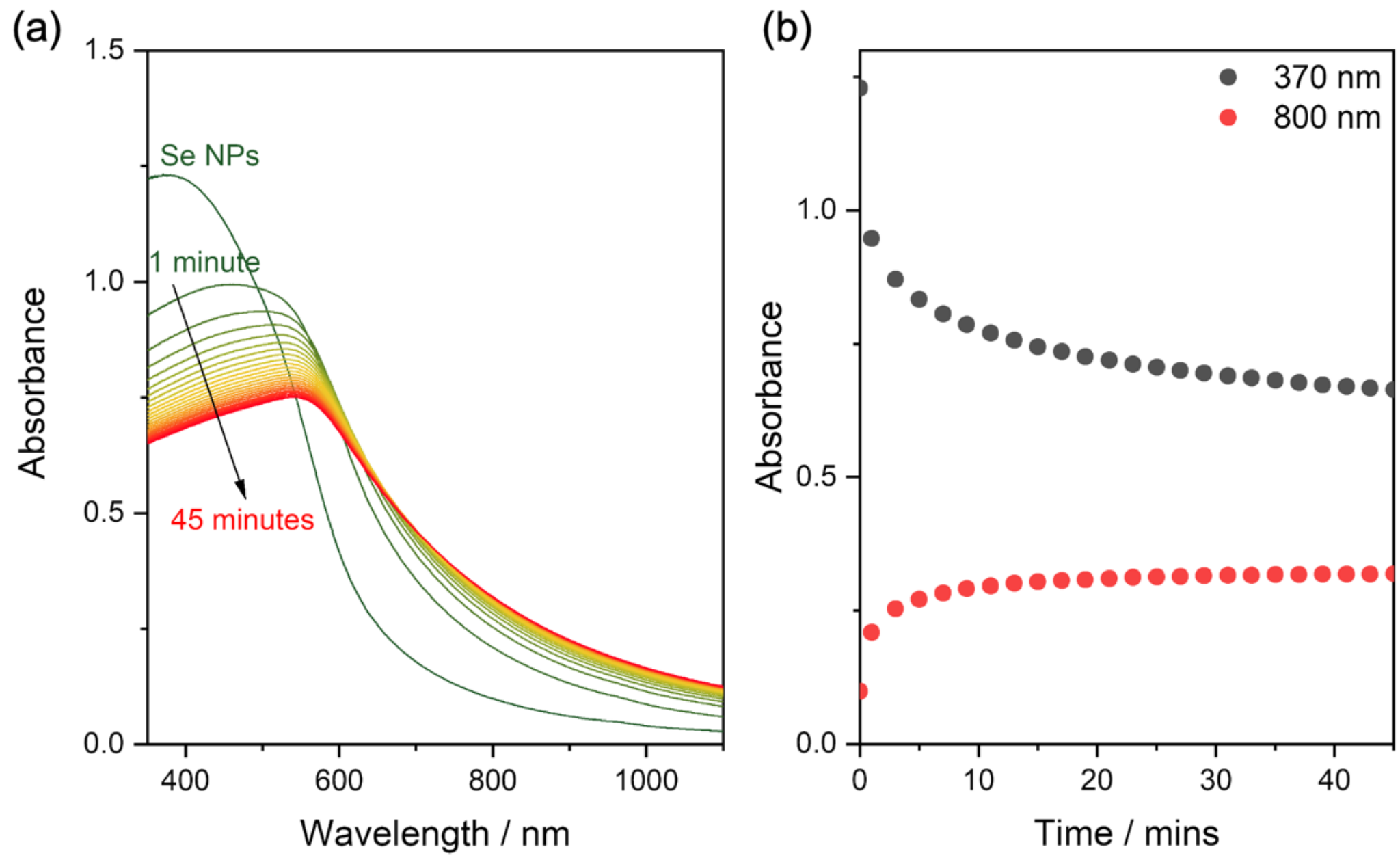

Fig. S4. (a) Absorbance of Se NPs undergoing aggregation caused by the addition of CB7 (final concentration = 1 µM) measured over 45 minutes (arrow indicates passage of time from Se NPs prior to CB7 addition to 45 minutes, every 2 minutes, after addition). (b) Isolated change in 370 nm and 800 nm absorbance over time.

Fig. S5-S6 Dynamic light scattering

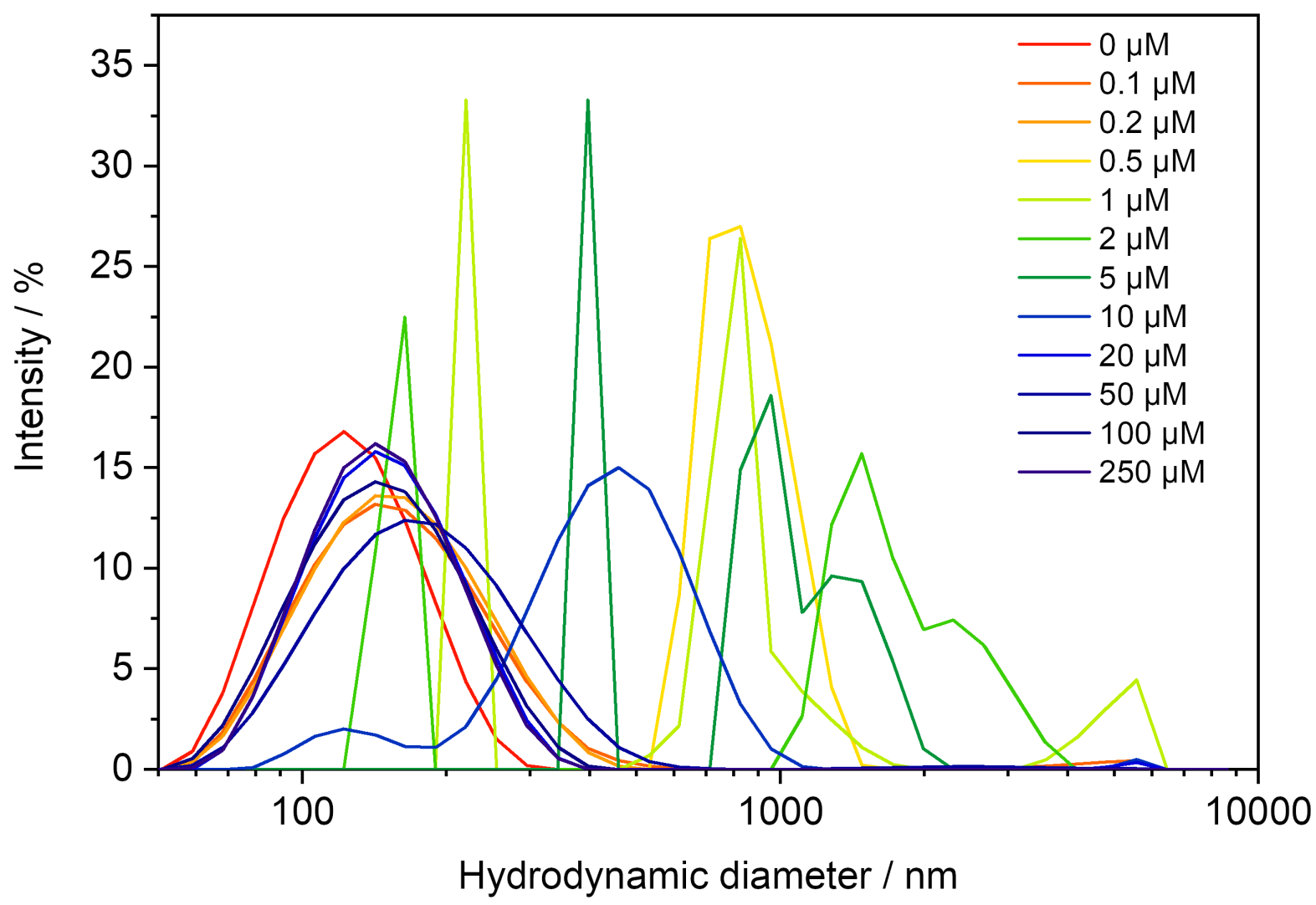

Fig. S5. Particle size distribution by intensity of Se NPs with varying amounts of CB7 (concentrations listed).

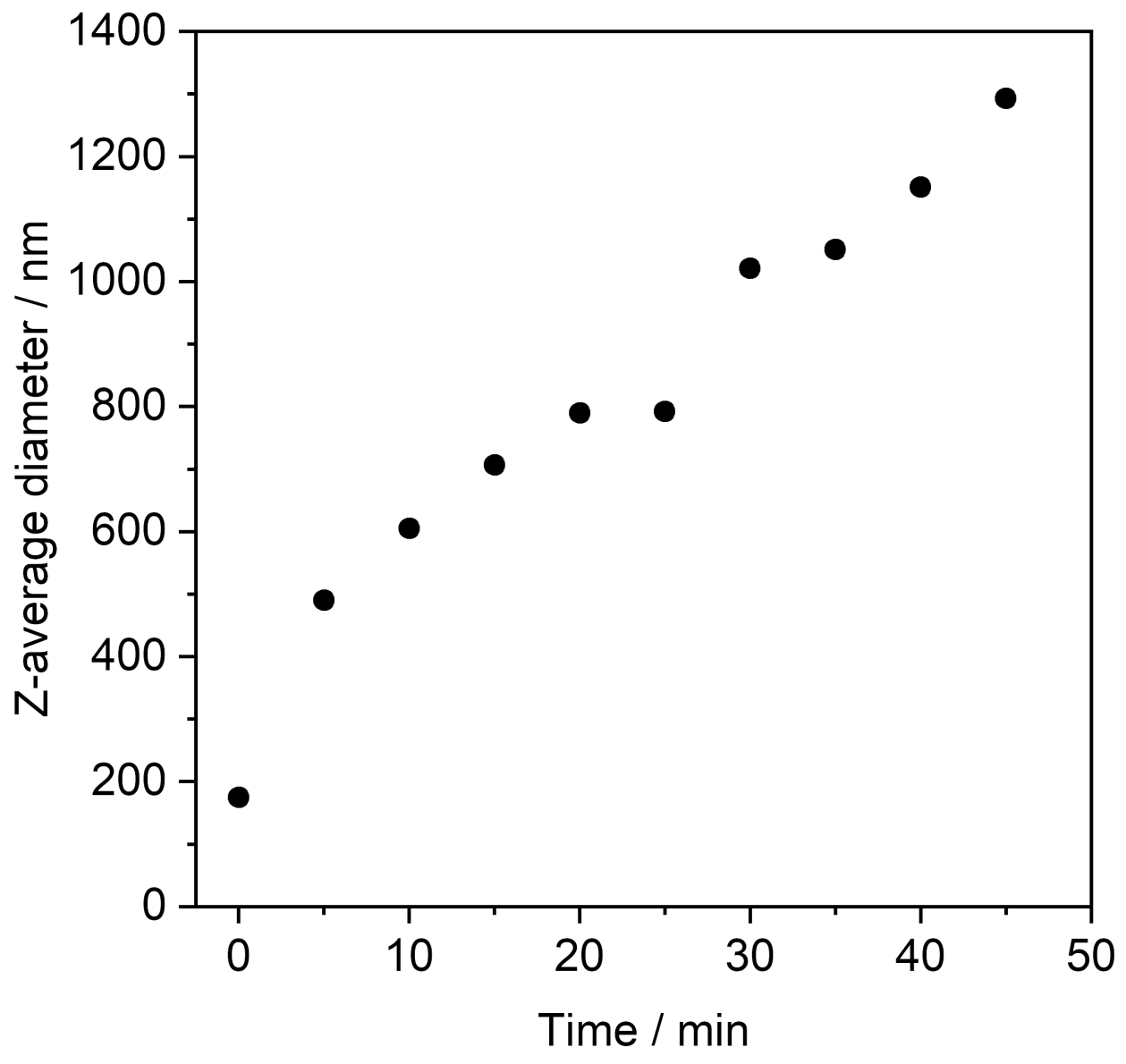

Fig. S6. Se NP aggregation caused by the addition of CB7 (final concentration = 1 µM) measured over 45 minutes.

Fig S7 Additional UV-visible spectroscopy

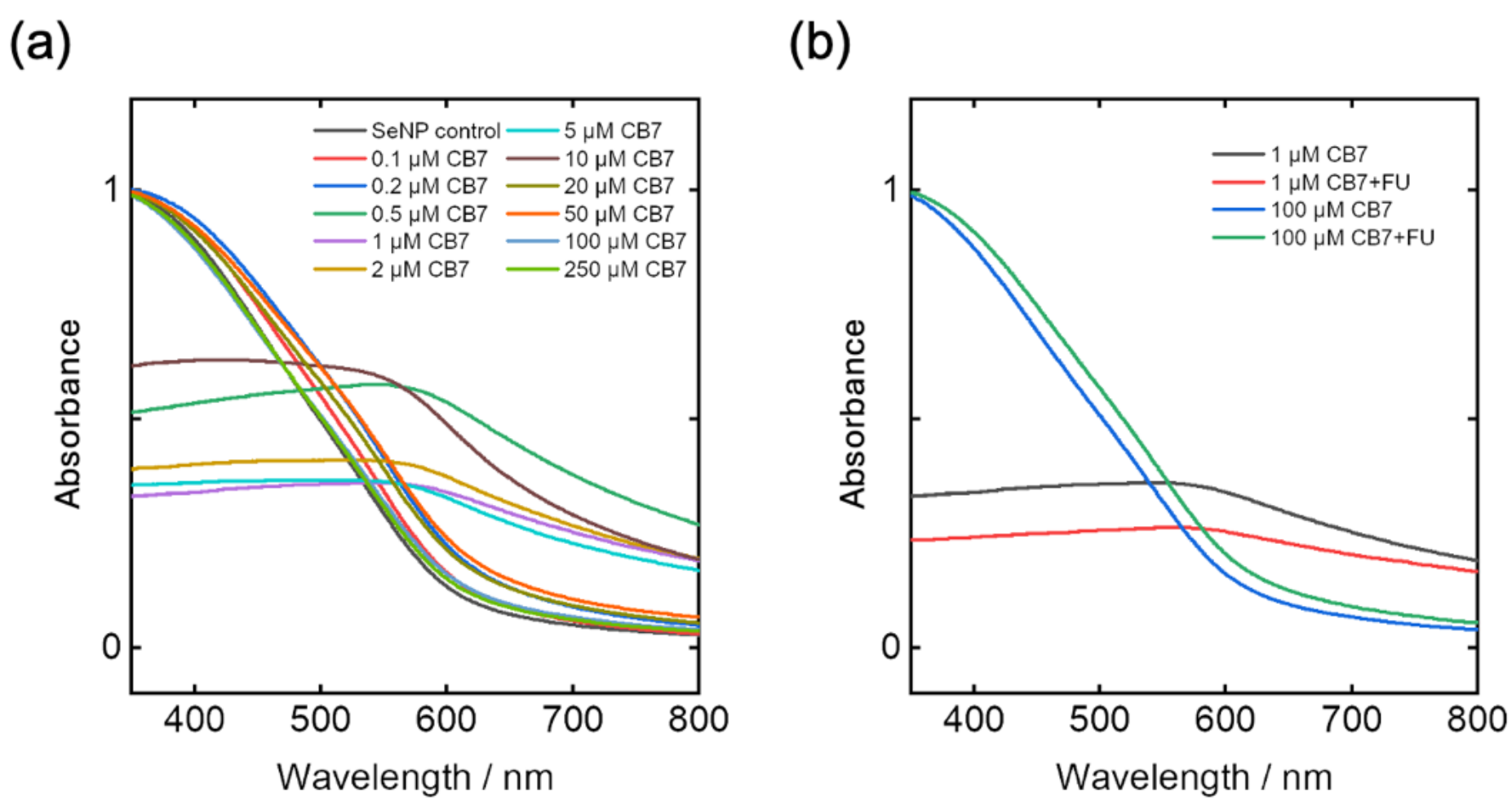

Fig. S7. a) UV-vis spectra of all Se NP+CB7 samples. b) UV-vis spectra of Se NP+CB7+FU samples.

Fig. S8-S9 Particle diameter/interparticle distance measurements

Digital Micrograph was used to determine particle size and interparticle spacing, using contrast-difference line profiles.

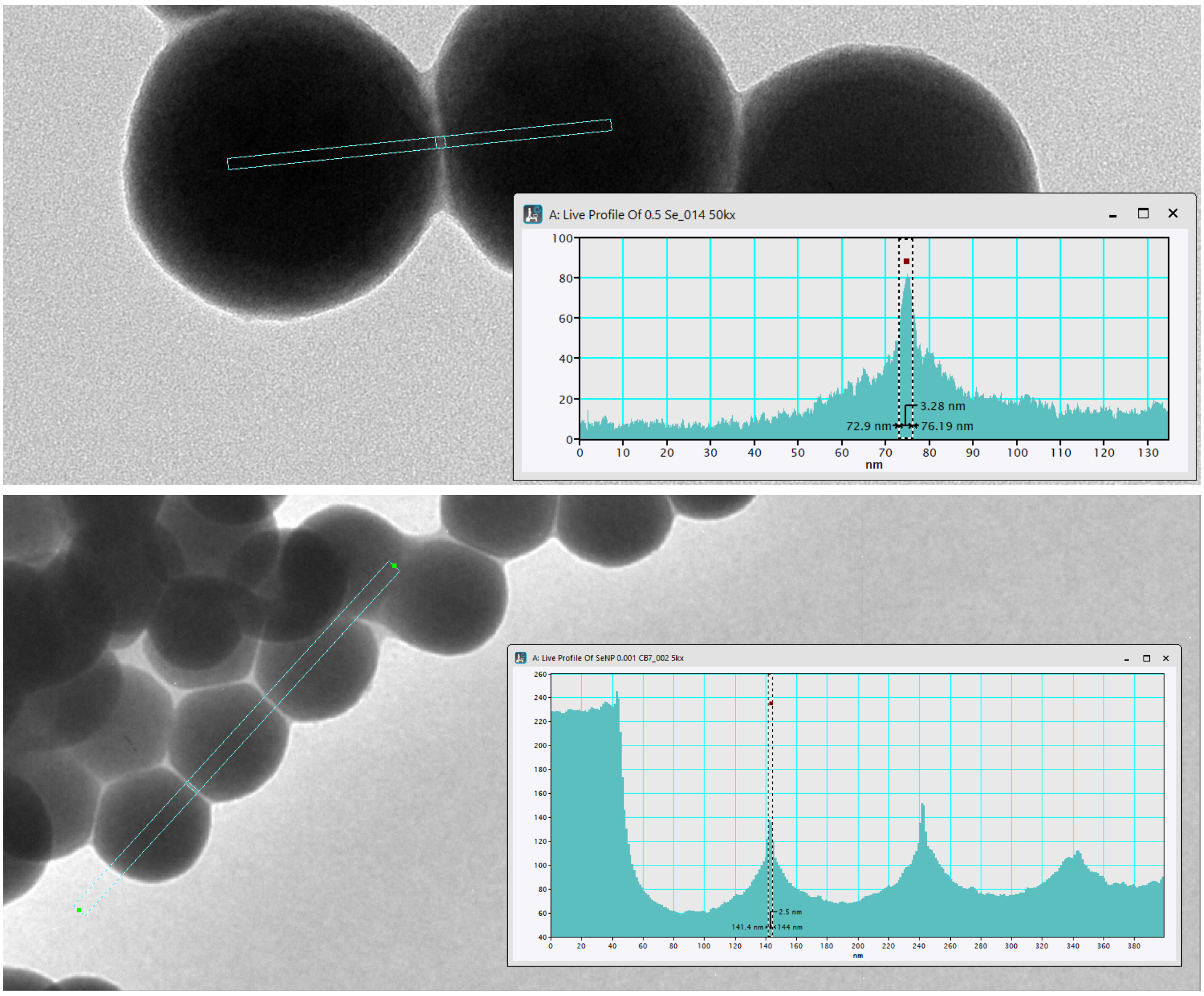

Fig. S8. Example of a contrast-difference line profiles used to determine interparticle spacing for Se NPs (above), and Se NPs with 1 µM CB7 (below).

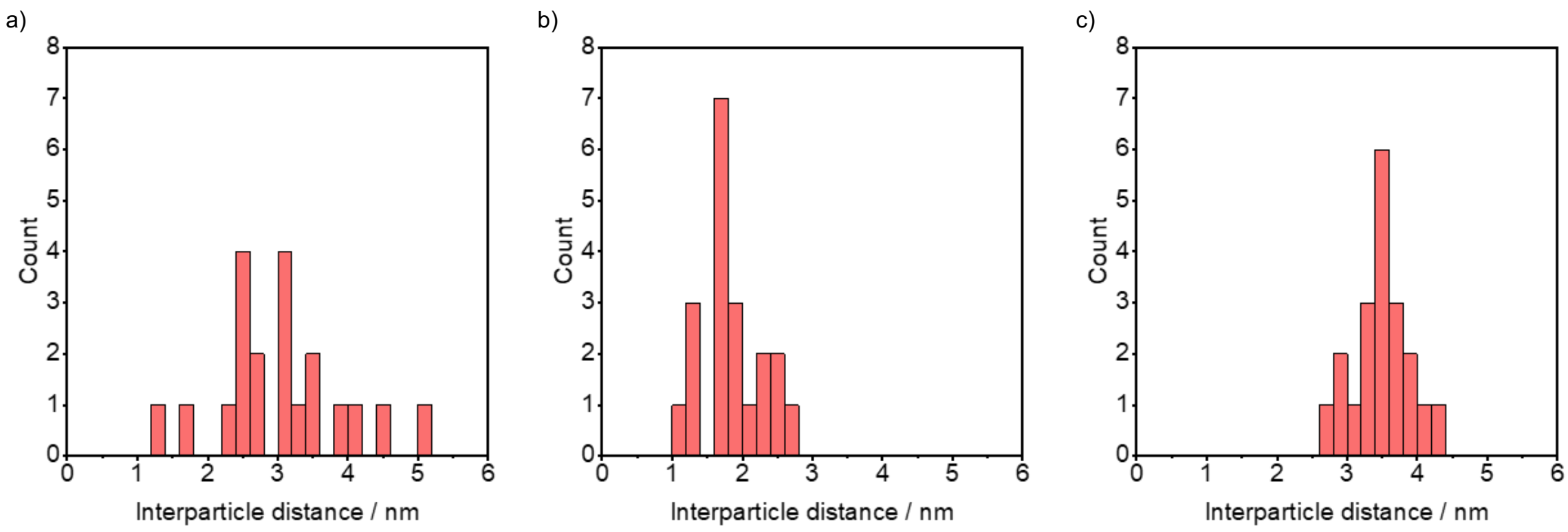

Fig. S9. Interparticle distances measured for Se NPs with a) 0 µM CB7, b) 1 µM CB7 and c) 250 µM CB7.

Fig. S10 Zeta potential measurements

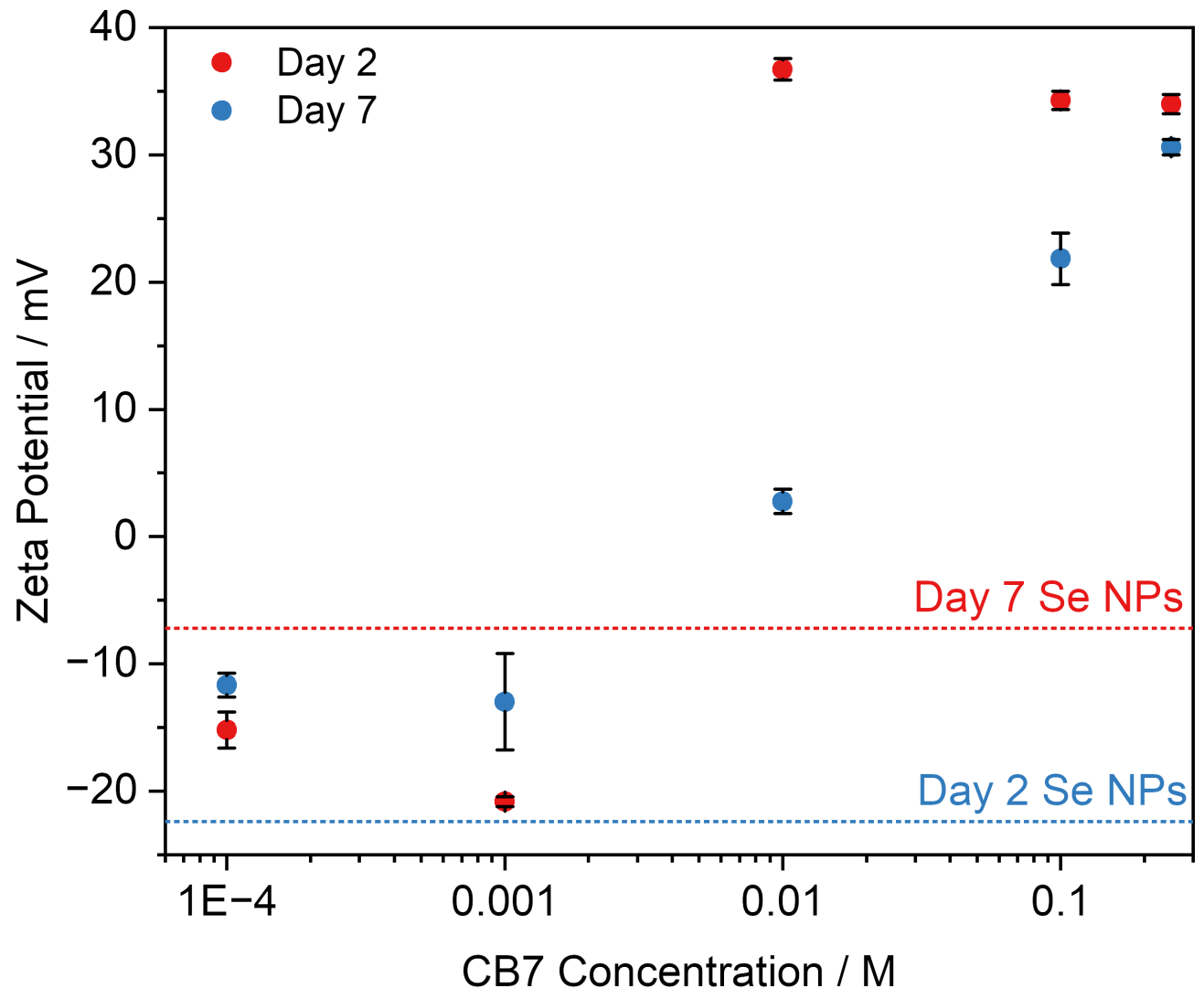

Fig. S10. Zeta potential changes for Se NPs caused by the addition of CB7 for Se NPs at day 2 and day 7. Dashed lines show the values for Se NPs with no CB7 added.

Fig S11 Full CB7-FU NMR

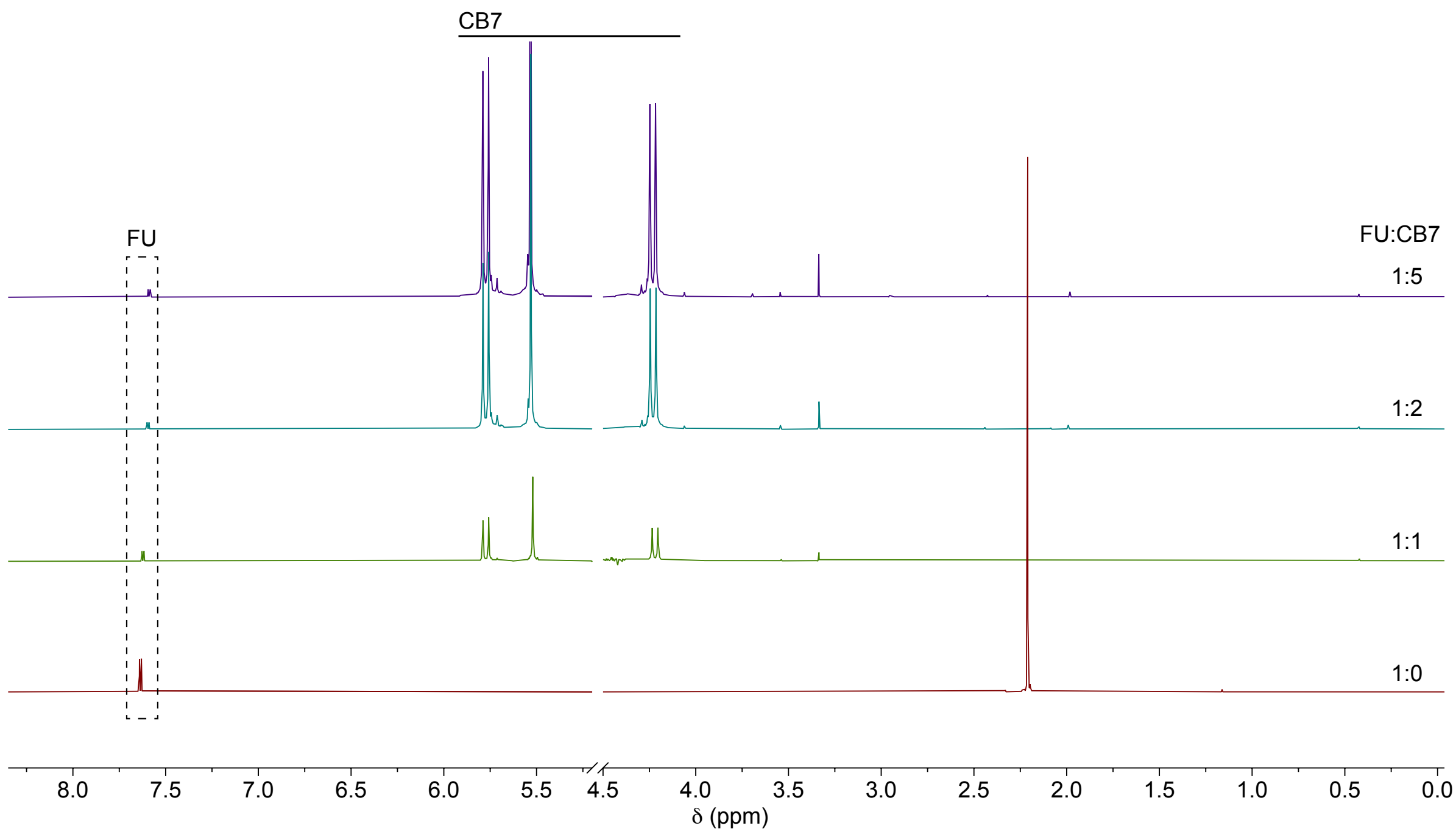

Fig. S11 Full ^{1}H NMR (D_2O, 500 MHz) spectra for CB7 and FU with FU held at 0.3 mM and 0 to 5 equivalents of CB7 added.

Fig. S12 DFT modelling

FU was docked within CB7 by screening binding poses using AutoDock Vina's search algorithm and scoring function, retrieving six unique binding positions.[3,4] Further geometry optimization and binding affinity calculations were carried out in Spartan 20 and cross validated by Gaussian 16 at the wB97XD/6-31G* level of theory.[6]

Gaussian 16 binding constant calculations were performed utilising a solvent model and applying thermal corrections. The solutes were placed within a cavity in a solvent interaction field by applying the polarizable conductor calculation model (CPCM): a continuum solvent model.[7,8] Thermochemical analysis using the rigid rotor harmonic oscillator model as implemented in Gaussian 16 determined vibrational frequencies, with subsequent calculation of translational, electronic, rotational, and vibrational free energy corrections. The binding constant log*K* for the ground state binding pose was calculated to be 6.30.

Similar binding calculations were carried out with GFN2-xTB, applying the Analytical Linearized Poisson Boltzmann solvent model (ALPB), and rigid rotor harmonic oscillator (RRHO) model to determine thermal corrections to free energy. The binding constant retrieved was 7.78, comparable to that obtained from Gaussian 16.

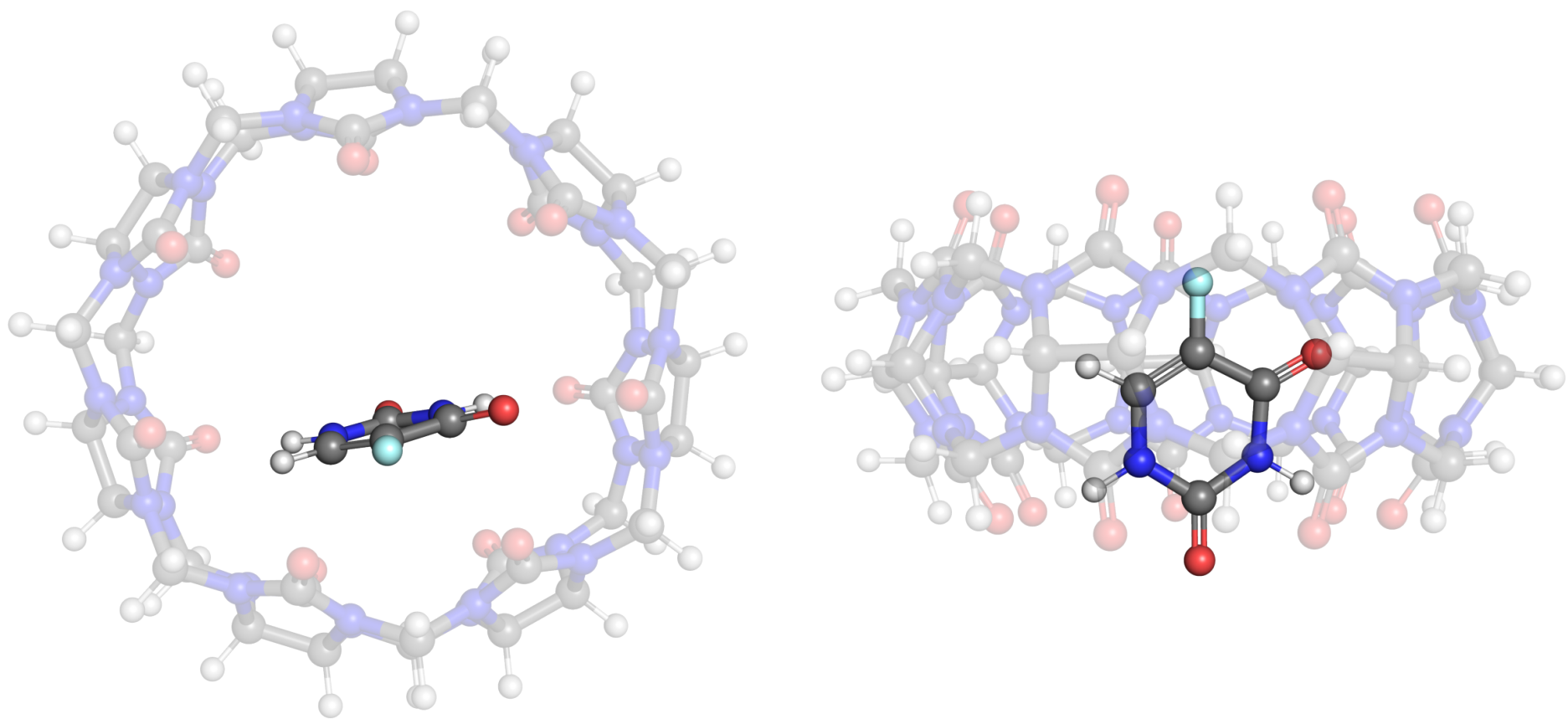

Fig. S12. Top and side perspectives of the ground state structure of the CB7-FU inclusion complex optimized at the wB97XD/6-31G* level.

Fig. S13 Additional NMR titrations

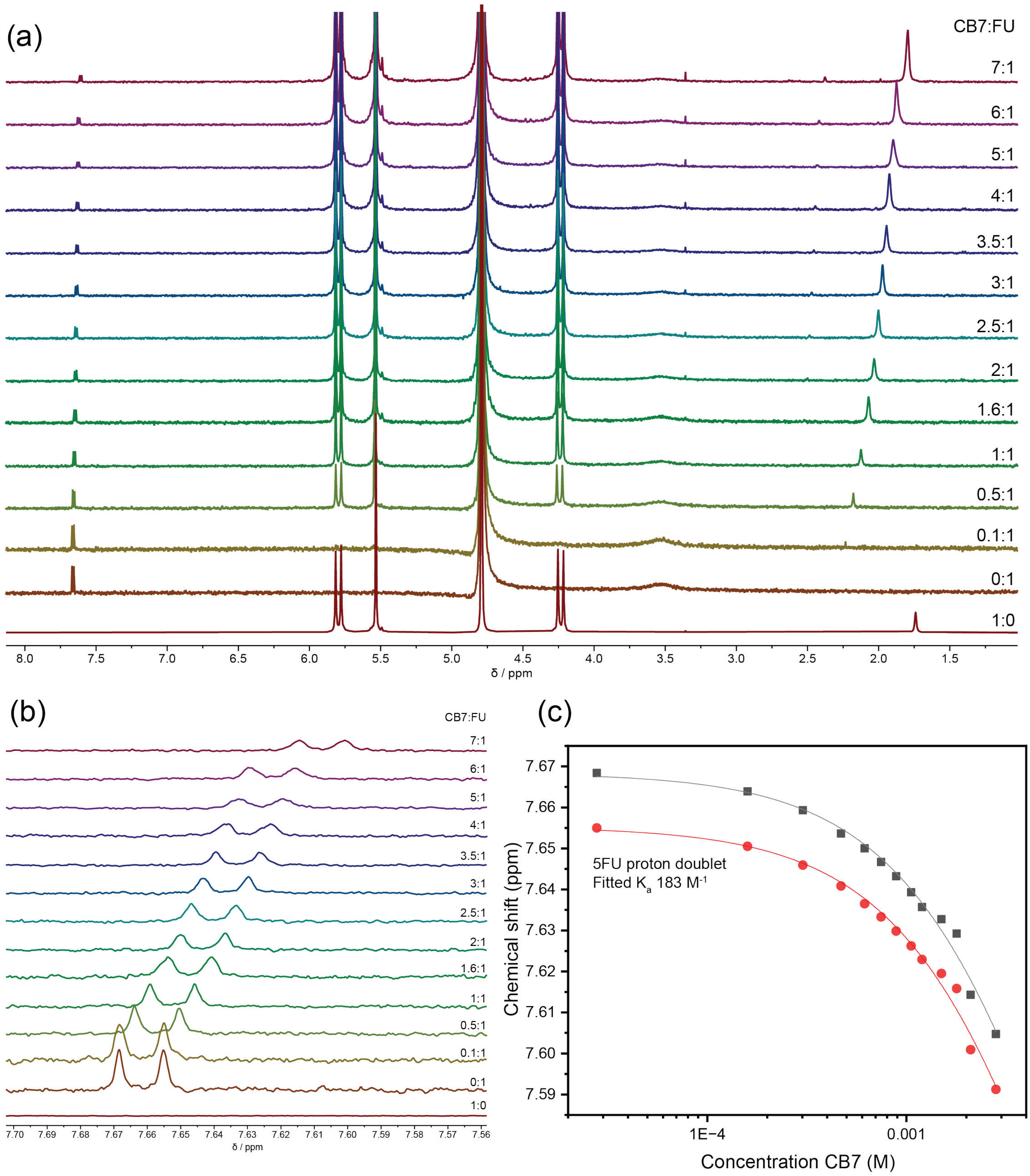

Fig. S13. An NMR titration as undertaken with an alternate batch of CB7 and FU. FU was held at 0.3 mM and the concentration of CB7 was increased (with additional FU also added to correct for dilution effects) up to a ratio of 7:1. The D_2O used was pH corrected to ~4 with 1M $HCl_{(aq)}$ to better match the Se NP solution, whilst noting that local pH may vary a lot at the NP surface. The same shifting of the FU aromatic doublet was observed and this was fitted to a 1:1 binding model, giving a K_a of 183 M^{-1}. However this value was very pH dependent and much smaller than the initial value found for the alternate batch of CB7, suggesting that solvent effects and CB batch can both impact the binding coefficient.

Fig. S14 Additional host-guest chemistry at Se NP surfaces

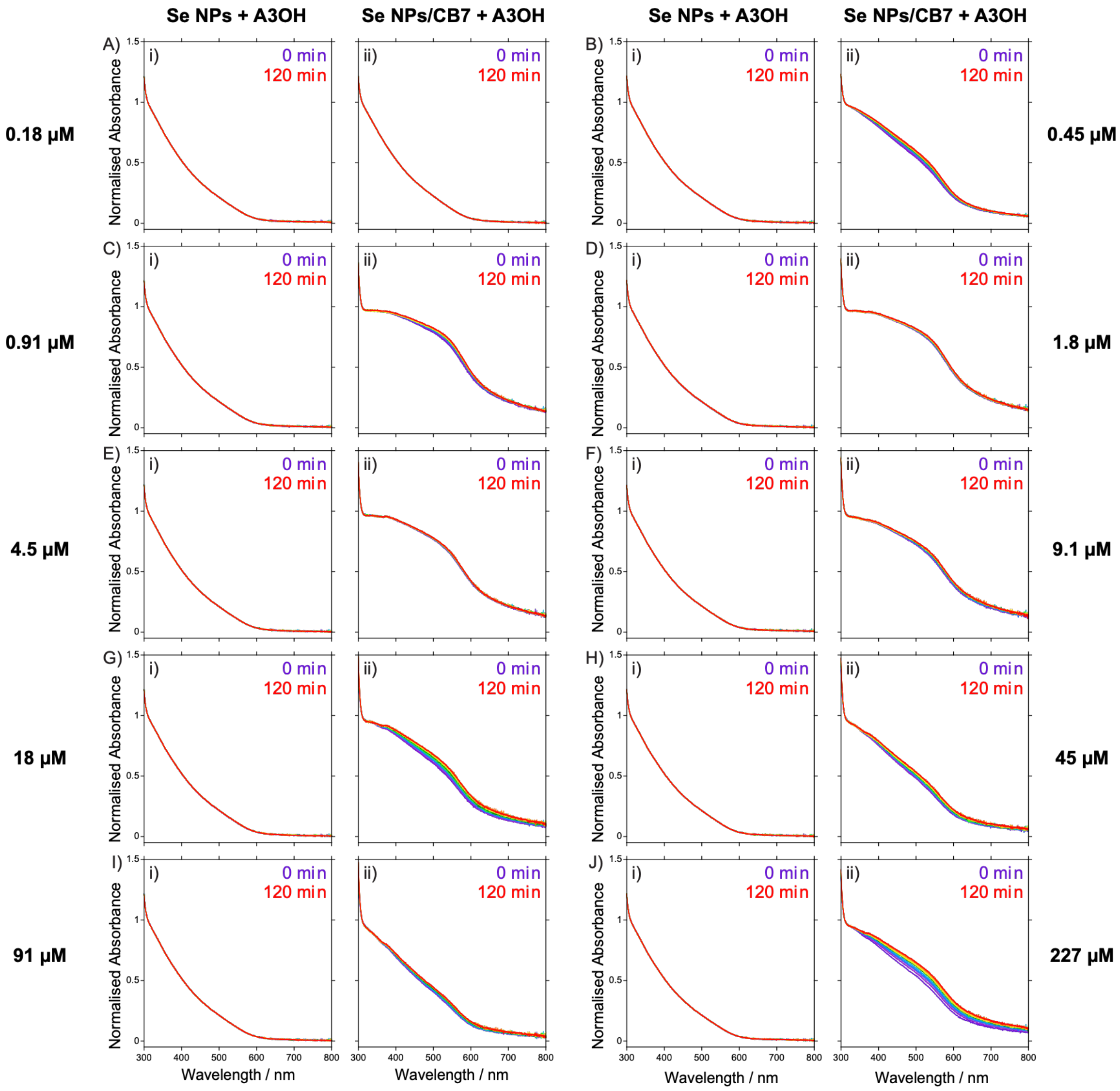

Fig S14a. Se NPs were mixed with i) A3OH and incubated for 2 hours, monitoring by UV vis; or ii) CB7 and incubated for two hours, before adding A3OH and monitoring with UV Vis for a further 2 hours. Spectra were recorded every 10 minutes and each is an average of 3 replicates. The bold numbers give the CB7 and/or A3OH concentration used in each case. Addition of A3OH to preformed aggregates does not easily break up the aggregates, and addition to Se NPs more completely coated in CB7 at higher concentrations appears to lead to a degree of increased aggregation. This implies that the short triethylene glycol tail is not long enough to fully stabilize the large Se NPs, that the inside of the CB7 cavity in aggregates is hard to access, and that the charge screening and lowering of the zeta potential may result in increased flocculation due to van der Waals interactions.

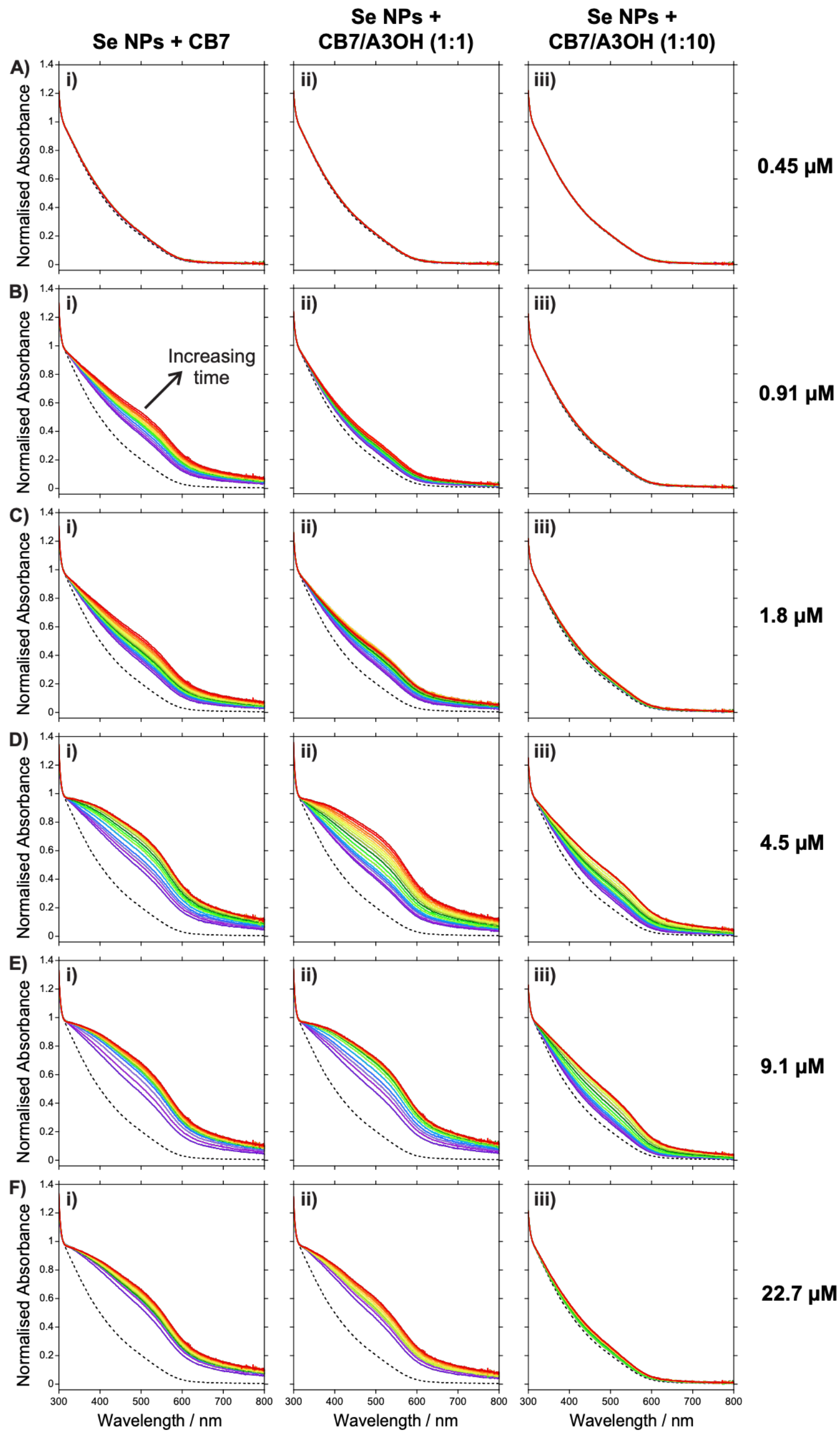

Fig S14b. Se NPs were mixed with CB7 (i), or CB7 premixed with one equivalent (ii), or 10 equivalents (iii) of A3OH. The high affinity of A3OH for CB7 should result in complex formation and the A3OH acts as a 'one portal binder',[2] so the Se NP surfaces should become coated with CB7 and A3OH. The bold numbers indicate the concentration of CB7 used in each case and the process was monitored by UV visible spectroscopy over 2 hours. The dashed lines are absorbance of the Se NPs alone. Adding A3OH to the process reduced the rate and overall amount of aggregation, but does not stop it completely, again implying that whilst the CB7 and A3OH can coat the Se NPs, the short ethylene glycol chain is not enough to offset the loss of surface charge and van der Waals forces can then lead to slow aggregation in some cases.

Fig. S15 Additional FTIR spectroscopy

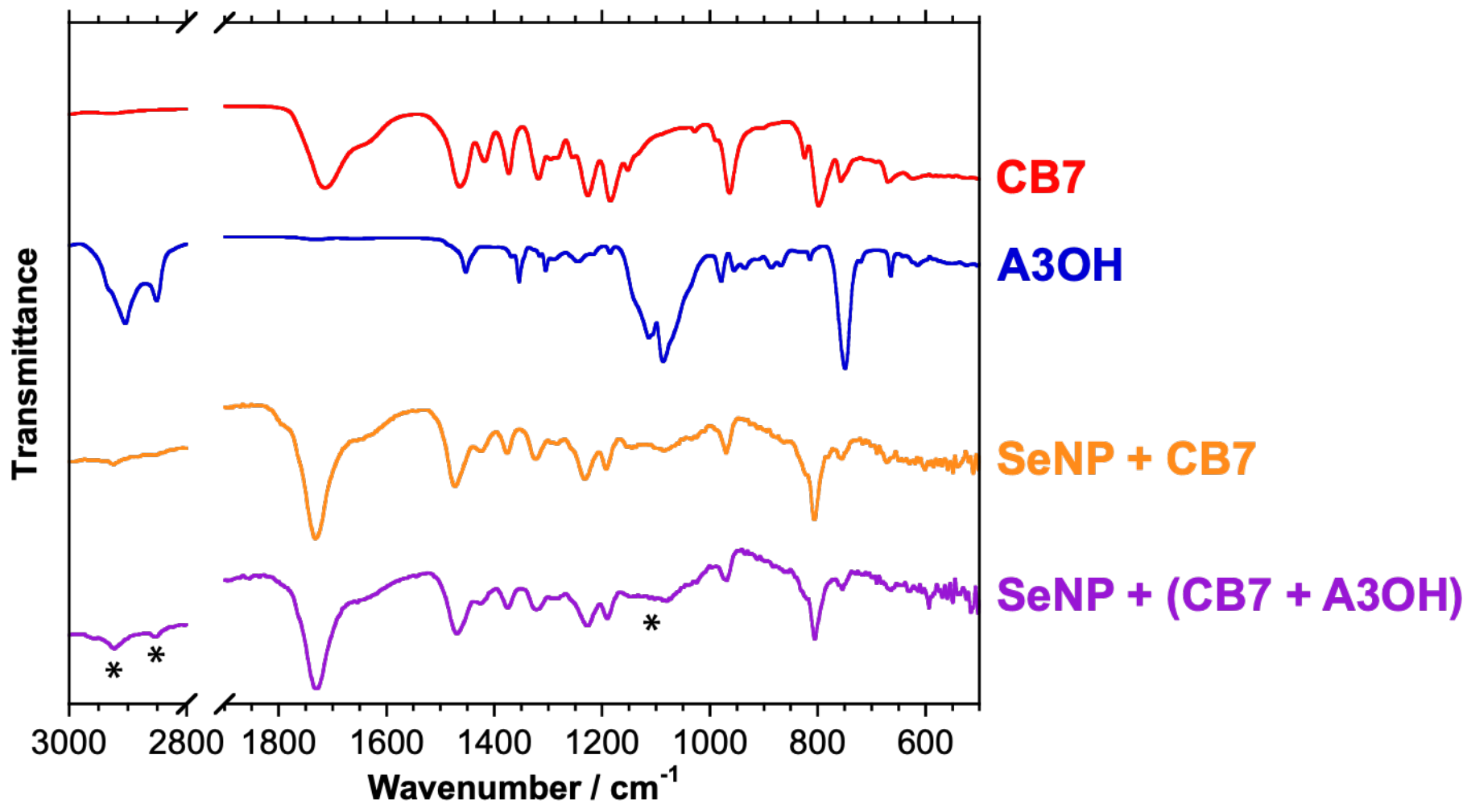

Fig. S15. FTIR of Se NPs with CB7 or with CB7 and A3OH, dried onto aluminium foil after centrifugal washing, showing indicators of A3OH presence along with the CB7 itself, with indicative peaks (*) at c. 2900 and 1100 cm^{-1}.

Fig. S16 Additional cytotoxicity data

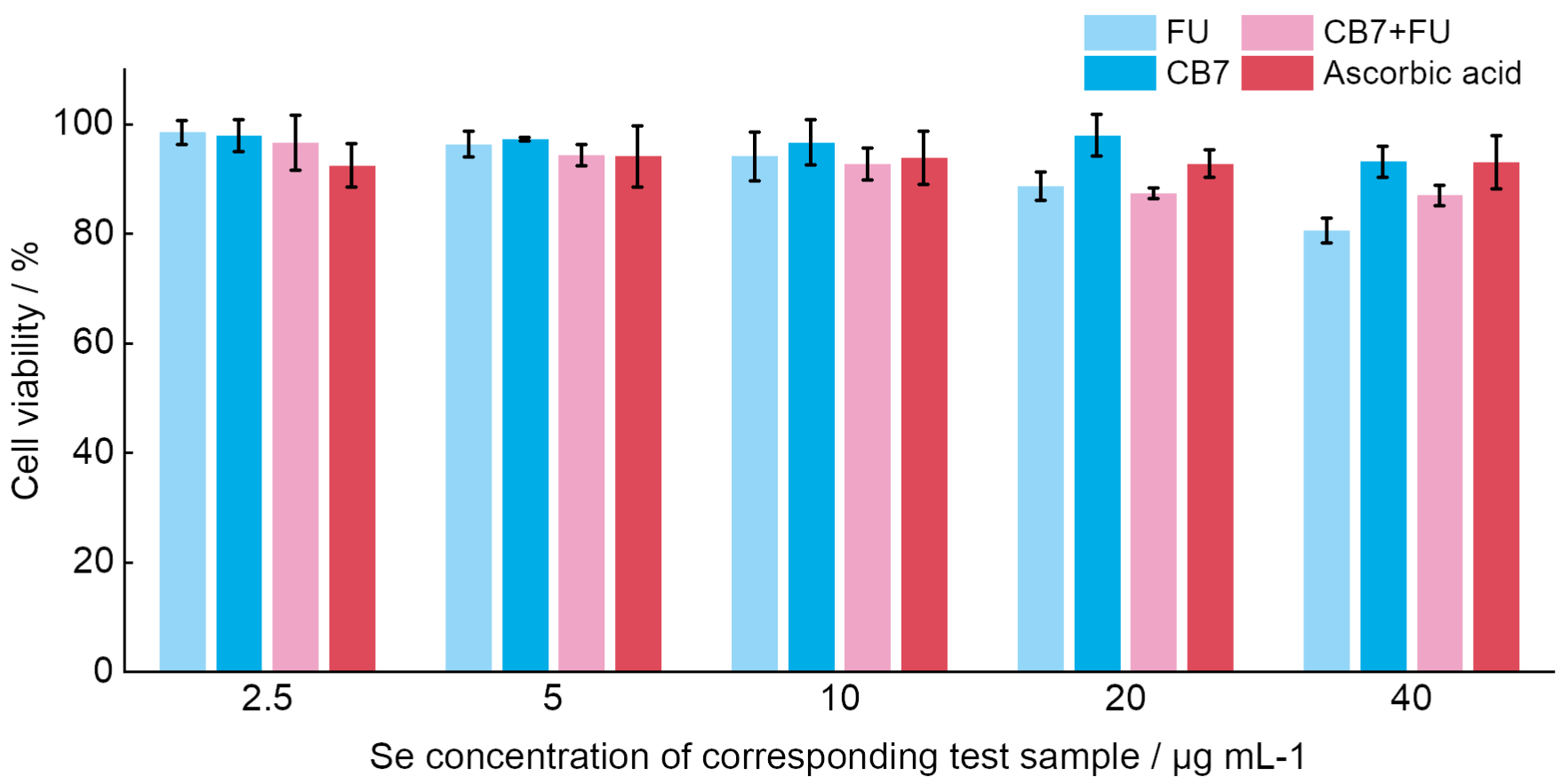

Fig. S16. Control tests for the activity of individual sample components against MCF7 cell viability after a 24 hour incubation period. Samples for this control test were prepared analogously to those which also included Se NPs, and the concentrations given are those of Se in the test samples which correspond to these control samples (no selenium is present in the control samples).

Table S1 Antimicrobial tests

Table S1. Minimum inhibitory concentrations (MIC) of Se NP samples against *E. Coli*.

Sample	MIC / μg mL^{-1}
Se NP	32
Se NP+CB7	32
Se NP+FU	16
Se NP+CB7+FU	16

References

1. W.-H. Huang, S. Liu and L. Isaacs, in Modern Supramolecular Chemistry, ed. F. Diederich, P. J. Stang and R. R. Tykwinski, Wiley-VCH, Weinheim, 2008, pp. 113–142.
2. K. McGuire, S. He, J. Gracie, C. Bryson, D. Zheng, A. W. Clark, J. Koehnke, D. J. France, W. M. Nau, T-C. Lee, W. J. Peveler, ACS Nano, 2023, 17, 21585–21594.
3. O. Trott and A. J. Olson, J. Comput. Chem., 2010, 31, 455–461.
4. J. Eberhardt, D. Santos-Martins, A. F. Tillack and S. Forli, J. Chem. Inf. Model., 2021, 61, 3891–3898.
5. C. Bannwarth, S. Ehlert and S. Grimme, J. Chem. Theory Comput., 2019, 15, 1652–1671.
6. M. J. Frisch, G. W. Trucks, H. B. Schlegel, G. E. Scuseria, M. A. Robb, J. R. Cheeseman, G. Scalmani, V. Barone, G. A. Petersson, H. Nakatsuji, X. Li, M. Caricato, A. V. Marenich, J. Bloino, B. G. Janesko, R. Gomperts, B. Mennucci, H. P. Hratchian, J. V. Ortiz, A. F. Izmaylov, J. L. Sonnenberg, D. Williams-Young, F. Ding, F. Lipparini, F. Egidi, J. Goings, B. Peng, A. Petrone, T. Henderson, D. Ranasinghe, V. G. Zakrzewski, J. Gao, N. Rega, G. Zheng, W. Liang, M. Hada, M. Ehara, K. Toyota, R. Fukuda, J. Hasegawa, M. Ishida, T. Nakajima, Y. Honda, O. Kitao, H. Nakai, T. Vreven, K. Throssell, J. A. Montgomery Jr., J. E. Peralta, F. Ogliaro, M. J. Bearpark, J. J. Heyd, E. N. Brothers, K. N. Kudin, V. N. Staroverov, T. A. Keith, R. Kobayashi, J. Normand, K. Raghavachari, A. P. Rendell, J. C. Burant, S. S. Iyengar, J. Tomasi, M. Cossi, J. M. Millam, M. Klene, C. Adamo, R. Cammi, J. W. Ochterski, R. L. Martin, K. Morokuma, O. Farkas, J. B. Foresman and D. J. Fox, Gaussian 16, Revision A.03, Gaussian, Inc., Wallingford CT, 2016.
7. V. Barone and M. Cossi, J. Phys. Chem. A, 1998, 102, 1995–2001.
8. M. Cossi, N. Rega, G. Scalmani and V. Barone, J. Comput. Chem., 2003, 24, 669–681.
